# Multi-proteins similarity-based sampling to select representative genomes from large databases

**DOI:** 10.1186/s12859-025-06095-3

**Published:** 2025-05-06

**Authors:** Rémi-Vinh Coudert, Jean-Philippe Charrier, Frédéric Jauffrit, Jean-Pierre Flandrois, Céline Brochier-Armanet

**Affiliations:** 1 Université Claude Bernard Lyon 1, LBBE, UMR 5558, CNRS, VAS, 69622 Villeurbanne, France; 2 Microbiology Research and Development, BioMérieux SA, 376 Chemin de L’Orme, 69280 Marcy-L’Étoile, France; 3 Institut Universitaire de France, Paris, France

**Keywords:** Genome dereplication, Dice index, Prokaryotes, RiboDB, GTDB, Genome collection

## Abstract

**Background:**

Genome sequence databases are growing exponentially, but with high redundancy and uneven data quality. For these reasons, selecting representative subsets of genomes is an essential step for almost all studies. However, most current sampling approaches are biased and unable to process large datasets in a reasonable time.

**Methods:**

Here we present MPS-Sampling (Multiple-Protein Similarity-based Sampling), a fast, scalable, and efficient method for selecting reliable and representative samples of genomes from very large datasets. Using families of homologous proteins as input, MPS-Sampling delineates homogeneous groups of genomes through two successive clustering steps. Representative genomes are then selected within these groups according to predefined or user-defined priority criteria.

**Results:**

MPS-Sampling was applied to a dataset of 48 ribosomal protein families from 178,203 bacterial genomes to generate representative genome sets of various size, corresponding to a sampling of 32.17% down to 0.3% of the complete dataset. An in-depth analysis shows that the selected genomes are both taxonomically and phylogenetically representative of the complete dataset, demonstrating the relevance of the approach.

**Conclusion:**

MPS-Sampling provides an efficient, fast and scalable way to sample large collections of genomes in an acceptable computational time. MPS-Sampling does not rely on taxonomic information and does not require the inference of phylogenetic trees, thus avoiding the biases inherent in these approaches. As such, MPS-Sampling meets the needs of a growing number of users.

**Supplementary Information:**

The online version contains supplementary material available at 10.1186/s12859-025-06095-3.

## Background

The burst of genome sequencing provides a wealth of data and an ever-increasing access to genetic information, including from uncultured organisms [1]. However, available genomic data, and especially genome sequences, are growing in an unbalanced way, both in terms of quality, as most released genomes are in fact rough draft assemblies, and diversity, with the over-representation of a few taxa, reflecting socio-economic considerations [2] (Additional files 1–2). In this context, exhaustive analyses of available genomes are complex, time-consuming, technically impossible and, in most cases, irrelevant. Therefore, most studies use subsamples of available genomes [2]. Current sampling strategies are usually based on taxonomic, phylogenic, or genome similarity criteria, but they all have the same objective: finding the best balance between sample size and representativeness, and proceed in the same way: the grouping of genomes, then the selection of representatives either at random [3] or according to a priori defined criteria (e.g., type strains, completeness) [4, 5].

Taxonomy-based approaches group genomes according to their taxonomy [3, 5]. Although easy to apply, these approaches have limitations. For instance, while they represent a significant part of the biodiversity (e.g. up to 60% in the study by Parks et al*.* [6]), many genomes have incomplete or even no taxonomic assignment (Additional file 2). Most of the time, these genomes are omitted, which can lead to important gaps. Furthermore, these approaches are sensitive to taxonomy errors [5] and hampered by historic legacy [7]. Phylogeny-based approaches seek to group genomes according to the information provided by a phylogenetic tree (e.g. tree topology, branch supports, patristic distances). Several tools have been developed, such as Treemmer [8], PhyCLIP [9], TreeCluster [10], or AncestralClust [11]. However, they often include manual curation steps, require taxonomic information, and cannot handle very large phylogenetic trees due to computational time and memory usage. Furthermore, the quality of trees decreases as the number and divergence of sequences increase [12], which may impact on the reliability of the genome clustering and thus of the sampling. Most genome similarity-based methods are based on the computation of Overall Genome Relatedness Indexes (OGRI) [13], such as the average nucleotide identity (ANI) [14] or the shared ratio of k-mers [15]. While being in principle taxonomically and phylogenetically agnostic, these approaches have also some limits. First, they are constrained by the quadratic complexity as they require exhaustive genome pairwise comparisons [13]. Second, the accuracy of OGRIs strongly decreases as the evolutionary divergence between genomes increases (i.e. above the species or genus levels) [16]. Finally, several international consortia provide their own sets of representative genomes (Additional file 3). However, these ready-to-use datasets are of limited interest as users have no control over the sampling density, redundancy, or data update, and in most cases the genomes of undescribed organisms are not included.

Overall, current approaches for selecting representative genomes have important limitations and are not well suited to handle large collections of genomes. In our view, an ideal approach should be (i) able to process very large datasets with acceptable computational time, (ii) independent of taxonomy and phylogeny, (iii) scalable, (iv) reproducible, and (v) allow users to define their own criteria for the selection of representative genomes. To address these needs, we developed MPS-Sampling (Multi Protein Similarity-based Sampling), a fast, scalable, and reliable method for selecting representative genomes. MPS-Sampling uses families of homologous proteins as input and returns genome samples of variable density that are representative of both the taxonomic and phylogenetic diversity of the original datasets.

## Implementation

### MPS-sSmpling workflow

**Fig. 1:**
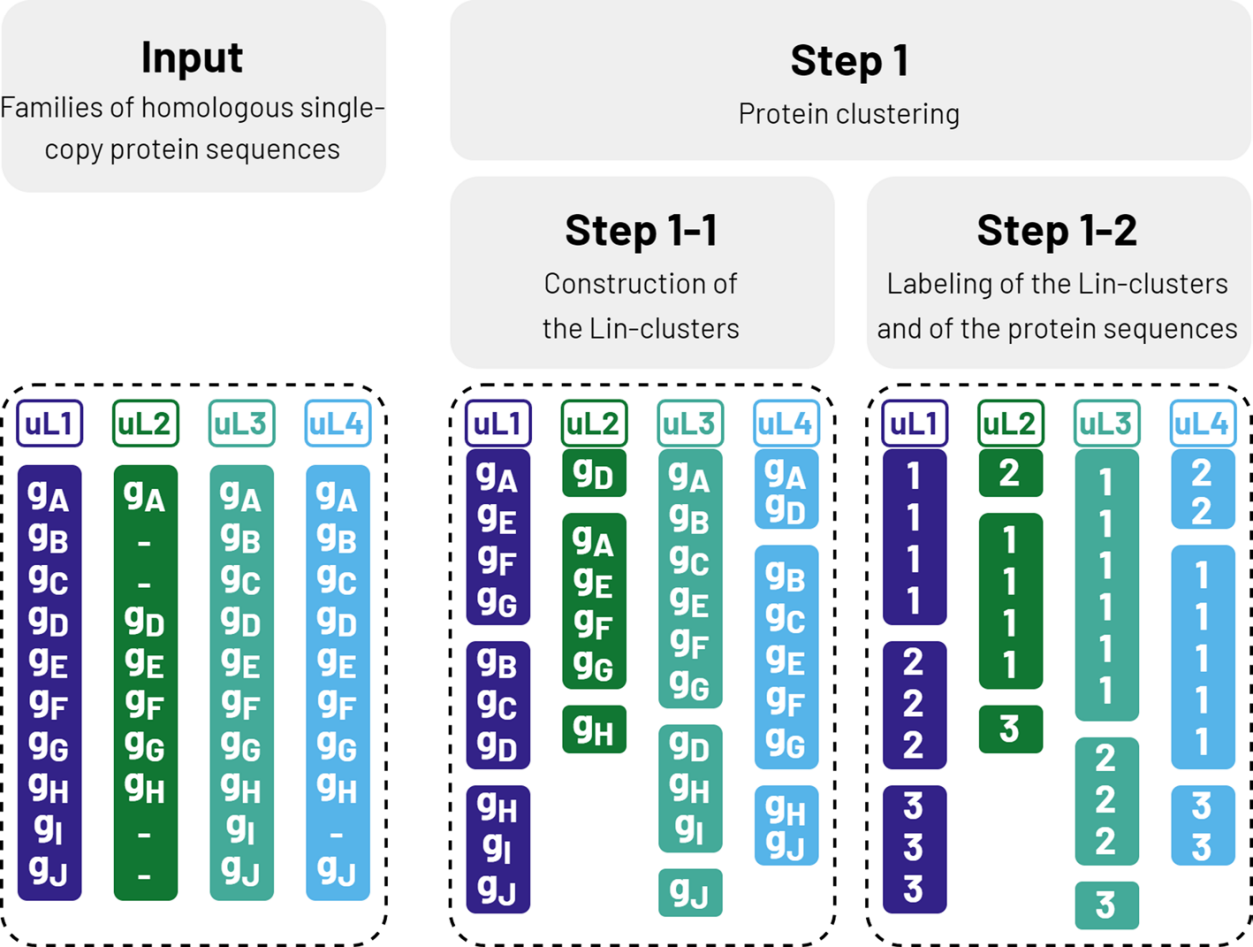
MPS-Sampling workflow: Input. MPS-Sampling uses families of homologous single-copy protein sequences as input. In this example, ten genomes (g_A_, g_B_, …, g_J_) and four protein families (uL1, uL2, uL3, and uL4) are considered. Dashes indicate the absence of a sequence in a genome. Here, uL2 is missing in g_B_, g_C_, g_I_ and g_J_, and uL4 is missing in g_I_. Step 1–1: Construction of Lin-clusters. For each protein family, sequence clusters, called Lin-clusters, are built using Linclust [17] of the MMseqs2 suite. Because Linclust is applied to each protein family, the clustering, and thus Lin-clusters, may differ from one protein family to another. Here, the uL1 sequence of g_A_ is clustered with uL1 sequences of g_E_, g_F_ and g_G_, while uL4 sequences of g_A_ and g_D_ are grouped together. Step 1–2: Labeling of protein sequences. For each protein family, protein sequences are labeled according to the Lin-cluster to which they belong. Sequences from the largest Lin-clusters are labeled first. For instance, uL1 sequences from the largest Lin-cluster (g_A_, g_E_, g_F_, and g_G_) are labeled as 1, sequences from the second largest Lin-cluster (g_B_, g_C_, and g_D_) as 2, while the third largest Lin-cluster (g_H_, g_I_, and g_J_) is labeled as 3

MPS-Sampling is distributed as a Snakemake pipeline (Additional file 4). The workflow is illustrated in Fig. 1, as well as in a flowchart (Additional file 5) and in an entity relationship diagram (ERD) (Additional file 6).

In brief.

- The input to MPS Sampling consists in a dataset of sequences organized into homologous families.
- Linclust [17] is applied to each protein family to cluster the sequences into Lin-clusters. Each Lin-cluster is labelled and each genome is described by a vector of $P$ Lin-cluster labels, where $P$ is the number of input protein families.
- Genomes with identical label vectors are grouped into elementary groups of genomes, called EGGs.
- Pairwise similarities are computed between genomes / EGGs using the Dice index. The similarity matrix is computed quickly using a one-hot encoding of the cluster labels.
- A complete-linkage hierarchical clustering is performed based on the Dice index values to build MPS-clusters.
- One representative genome, called the MPS-representative, is selected within each MPS-cluster. The set of MPS representatives corresponds to the sampling given by MPS-Sampling.

#### Input

MPS-Sampling uses families of unaligned homologous proteins as input (e.g. ribosomal families, core protein families) (Fig. 1). MPS-Sampling can handle missing data, meaning that protein families are not expected to be present in all genomes. The order of input data (i.e. genomes or protein families) does not affect the sampling results.

##### Step 1: construction of Lin-clusters

This step aims at identifying pairs of very similar sequences within families (step 1–1, Fig. 1). More precisely, within each protein family, sequences are clustered using Linclust of the MMseqs2 suite [17]. Linclust was chosen for its efficiency, very high specificity, high sensitivity, and near-linear complexity which allows to efficiently process very large datasets. Linclust identifies putative pairs of sequences through a kmer-based heuristic. The relevance of each pair is then evaluated by Linclust based on sequence alignment using three parameters: alignment e-value (eValue), coverage (minCov), and sequence identity (minSeqID). Linclust uses confirmed links to build sequence clusters, hereafter called Lin-clusters, according to the greedy set cover algorithm [18]. Lin-clusters are numbered from largest to smallest; the Lin-cluster encompassing the largest number of sequences being designated as 1 (step 1–2, Fig. 1).

##### Step 2: construction of elementary groups of genomes

**Fig. 2:**
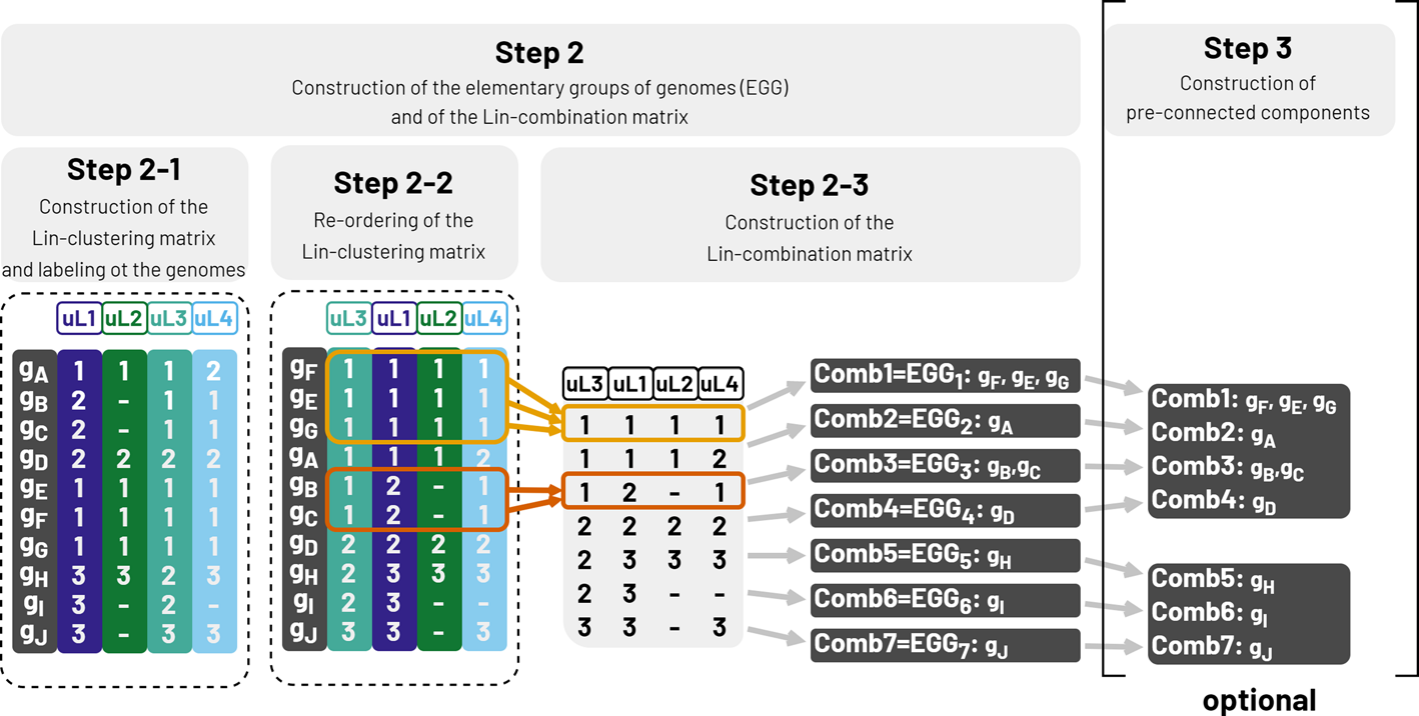
MPS-Sampling workflow: First clustering(s) of the genomes. Step 2–1: Construction of the Lin-clustering matrix and genome labeling. Lin-cluster labels are stored in a matrix, called the Lin-clustering matrix, whose rows correspond to genomes and columns to protein families. Here, genome g_A_ (first row) is labeled as (1, 1, 1, 2). Step 2–2: Re-ordering of the Lin-clustering matrix. To ensure reproducibility of the sampling, protein families (columns) and genomes (rows) are re-ordered. Protein families are ordered first according to the number of Lin-clusters they contain and then to the hashing value of their name. Here, all the four protein families have 3 Lin-clusters, thus are ordered according to their hashing value, giving the new order: uL3, uL1, uL2, uL4. Then, the genomes are ordered according first to the lexicographic order of their label, then to the hashing value of their name, giving the new order: g_F_, g_E_, g_G_, g_A_, g_B_, g_C_, g_D_, g_H_, g_I_, g_J_. Step 2–3: Construction of the Lin-combination matrix. Redundant lines of the Lin-clustering matrix are fused, leading to a smaller matrix, called the Lin-combination matrix, whose rows correspond to unique Lin-combinations of Lin-clusters. In this example, genomes g_B_ and g_C_ harbor the same Lin-combination (1, 2, -, 1) and are thus grouped together in the same Lin-combination (Comb3). At this step, the absence of sequences is not taken into consideration; for example, the absence of uL2 in g_B_ and g_C_ is not considered as a difference to separate these two genomes. The genomes grouped together into the same Lin-combination form an elementary group of genomes (EGG), because they are indistinguishable according to the parameters used during the run. Step 3: Construction of pre-connected components (optional). Lin-combinations are gathered into rough groups called pre-connected components, delineated during the pre-connection (Additional file 7). In the example, using minNbLinclusters = 2, two pre-connected components are built. A first pre-connected component gathers Comb1 (g_F_, g_E_, g_G_), Comb2 (g_A_), Comb3 (g_B_, g_C_), and Comb4 (g_D_), corresponding to seven genomes (g_F_, g_E_, g_G_, g_A_, g_B_, g_C_, and g_D_), while the second pre-connected component gathers Comb5 (g_H_), Comb6 (g_I_),and Comb7 (g_J_), encompassing three genomes (g_H_, g_I_, and g_J_)

This first clustering aims to gather close genomes into elementary groups of genomes (EGG). Each genome is associated to a vector of $P$ Lin-clusters, corresponding to those to which its sequences belong. These constitute the label of the genome, which is stored in the Lin-clustering matrix (step 2–1, Fig. 2). To ensure reproducibility, the Lin-clustering matrix is reordered by columns (protein families) and by rows (genomes) (step 2–2, Fig. 2). Genomes with the same label are then grouped together in the same EGG. The Lin-clustering matrix is reduced by removing identical lines, leading to a smaller and non-redundant matrix of labels, called the Lin-combination matrix (step 2–3, Fig. 2). Each line in the Lin-combination matrix corresponds to one EGG and contains at least one genome. This delineation is very strict, as genomes that differ by only one Lin-cluster are placed in separate EGGs. Within a given EGG, genomes are considered indistinguishable from this stage.

##### Step 3: construction of pre-connected components (optional)

This optional step aims at reducing the computation time by pre-defining rough groups of EGGs, called pre-connected components, prior the final clustering (step 3, Fig. 2 and Additional file 7).

##### Step 4: construction of MPS-clusters

**Fig. 3:**
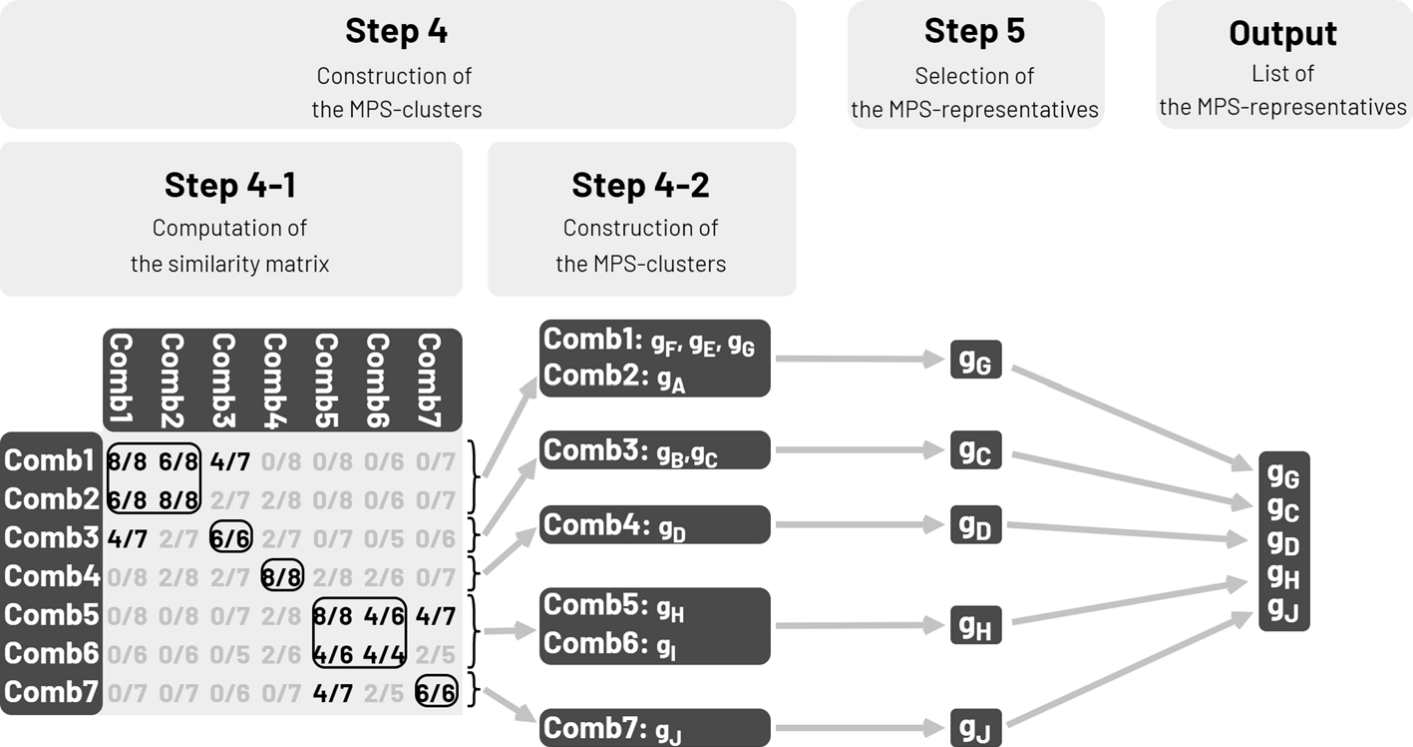
MPS-Sampling workflow: Final clustering of the genomes. Step 4–1: Computation of the similarity matrix. The similarity between each pair of Lin-combinations is computed and stored in a square matrix, called the similarity matrix (Additional file 9). The similarity is expressed by the Dice index [24] , which corresponds to the proportion of Lin-clusters shared by two Lin-combinations (missing values are omitted) (Additional file 8). Here, Comb6 and Comb7 share two Lin-clusters out of five (step 4-1, Fig. 3), so their Dice index in the matrix is 2/5. Step 4–2: Construction of MPS-clusters. The Lin-combinations (and the corresponding genomes) are clustered into MPS-clusters according to a hierarchical method with complete-linkage down to a minimum similarity Δ. In the example, five MPS-clusters are built using minimum similarity Δ = 0.5. A first MPS-cluster gathers two Lin-combinations: Comb1 (g_F_, g_E_, g_G_) and Comb2 (g_A_), corresponding to four genomes (g_F_, g_E_, g_G_, and g_A_). A second MPS-cluster gathers two Lin-combinations: Comb5 (g_H_) and Comb6 (g_I_), corresponding to the two genomes (g_H_ and g_I_). A third MPS-cluster encompasses only one Lin-combination Comb3 (g_B_ and g_C_), corresponding to two genomes (g_B_ and g_C_). The two last genomes corresponding to Comb4 (g_D_) and Comb7 (g_J_) are isolated and correspond to singleton MPS-clusters. Step 5: Selection of MPS-representatives. One MPS-representative genome is selected per MPS-cluster. These MPS-representatives are selected according to rigorous priority rules (Additional file 10). Here, g_G_, g_C_, g_D_, g_H_, and g_J_ are selected, each representing one MPS-cluster. Output: MPS-Sampling returns the list of the MPS-representative genomes, as well as the correspondance between each input genome and its MPS-representative genome

This second clustering aims at gathering close EGGs into groups, called MPS-clusters. The similarity between two EGGs is measured by the Dice index [19] (Additional file 8). Dice indexes are computed between all pairs of EGGs (or between pairs of EGGs within pre-connected components) and stored in the similarity matrix (or into the similarity submatrix of each pre-connected component) (step 4–1, Fig. 3). To avoid quadratic complexity, the matrix is calculated per column (Additional file 9). Then, EGGs, with a Dice index lower than a threshold called minimum similarity (Δ), are grouped according to an aggregative hierarchical method with complete linkage (step 4–2, Fig. 3). Δ varies from 0 to 1. Setting Δ to 1 is equivalent to bypassing this step of clustering and, in this case, MPS-clusters correspond to EGGs.

##### Step 5: selection of MPS-representatives

This step consists in the selection of one representative genome, called MPS-representative, within each MPS-cluster (Step 5, Fig. 3). The choice of the MPS-representatives respects rigorous priority rules, encompassing in the following order: the user-defined priority score, protein family distribution, centrality, and pseudo-randomization (Additional file 10).

#### Output: list of MPS-representatives

MPS-Sampling returns the list of MPS-representative genomes, as well as all intermediate results (e.g. the Lin-clustering matrix, the Lin-combination matrix, the similarity matrix, the link between each input genome and its MPS-representative) (Fig. 3).

### Datasets

MPS-Sampling was tested on the bacterial sequences of RiboDB v15.0 (Feb 2023), a dedicated database gathering ribosomal proteins (**r-prots**) [20]. This dataset, referred as to the bacterial dataset, encompasses 8,315,939 sequences from 48 r-prot families and 178,203 genomes (Additional file 12). More precisely, 157,405 (88%) genomes are from RefSeq, among which 16,135 (9%) are labeled as RefSeq-representatives by the NCBI, while other genomes are from Genbank. According to the NCBI, 135,315 genomes (76%) have complete taxonomic information, meaning that each relevant taxonomic level (i.e. phylum, class, order, family, genus, and species) is defined. MPS-Sampling was also tested on the data of the GTDB v214.1 of June 9th 2023, encompassing 120 core protein families present in 394,932 bacterial genomes [21]. To test MPS-sampling on a larger dataset, we simulated an artificial bacterial dataset (ABD) of 534,609 genomes (see Additional file 13).

### MPS-sampling parameters

MPS-Sampling parameters were optimized according to a standardized process (Additional files 14–21) and set as follow: eValue = 10^–5^, coverageMode = 0, minCov = 0.8, minSeqID = 0.6, and eleven values of Δ ∈ {1, 0.9, 0.8, 0.7, 0.6, 0.5, 0.4, 0.3, 0.2, 0.1, 0.05}.

### Taxonomic and phylogenetic diversity of the samples

For each sample, a phylogenetic tree was inferred (Additional file 22). The taxonomic diversity is measured by the proportion of phyla, classes, orders, families, genera, and species retained in the sample, the taxonomic redundancy by the average number of genomes kept per taxon, and the phylogenetic diversity by the length of the tree (i.e. the sum of all branch lengths) divided by the number of tips. Phylogenetic tree figures were drawn using iTOL v6.8.1 [22].

### Comparison with other tools

MPS-Sampling was compared with two other tools: Treemmer and TaxSampler. Treemmer allows to define groups of genomes according to phylogenetic criteria [8], while TaxSampler is a homemade program allowing to sample genomes at a given taxonomic level (Additional file 23).

## Results

### Computation time and memory usage

MPS-sampling was tested as a single-threaded process on a dedicated server with 32 cores and 64 threads (AMD EPYC 7542 32 Core Processor @3,40Ghz) and 1 To of DDR4 under Debian Trixie (Additional files 24-25A). MPS-Sampling computation time depends mainly on the size of the dataset (i.e. number of genomes, protein families). Without pre-connection, it required 1 h (elapsed time) to generate one sample from the bacterial dataset (178,203 genomes, 48 protein families). However, as steps 1 to 4–1 are common to all samplings and executed once, each additional sample only requires 4 min and the eleven runs required in total 1h41 . Regarding memory usage, MPS-Sampling requires 112 Gigabytes of memory usage to analyze the bacterial dataset (178,203 genomes, 48 protein families). As expected, the use of pre-connection significantly decreased computation time, from 1 h to 17 min (Additional file 25B). MPS-Sampling (without pre-connection) is 287 times faster than Treemmer, which required 360 h to generate one sample from the bacterial dataset and 1,992 h to generate eleven samples (Additional file 25C). For the ADB dataset (534,609 genomes, 48 protein families), MPS-Sampling required 10h55 to generate one sample (Additional file 25D), while it required 19h17 for the GTDB dataset (394,932 genomes, 120 protein families, not shown).

### Taxonomic and phylogenetic relevance of MPS-sampling samples

**Fig. 4:**
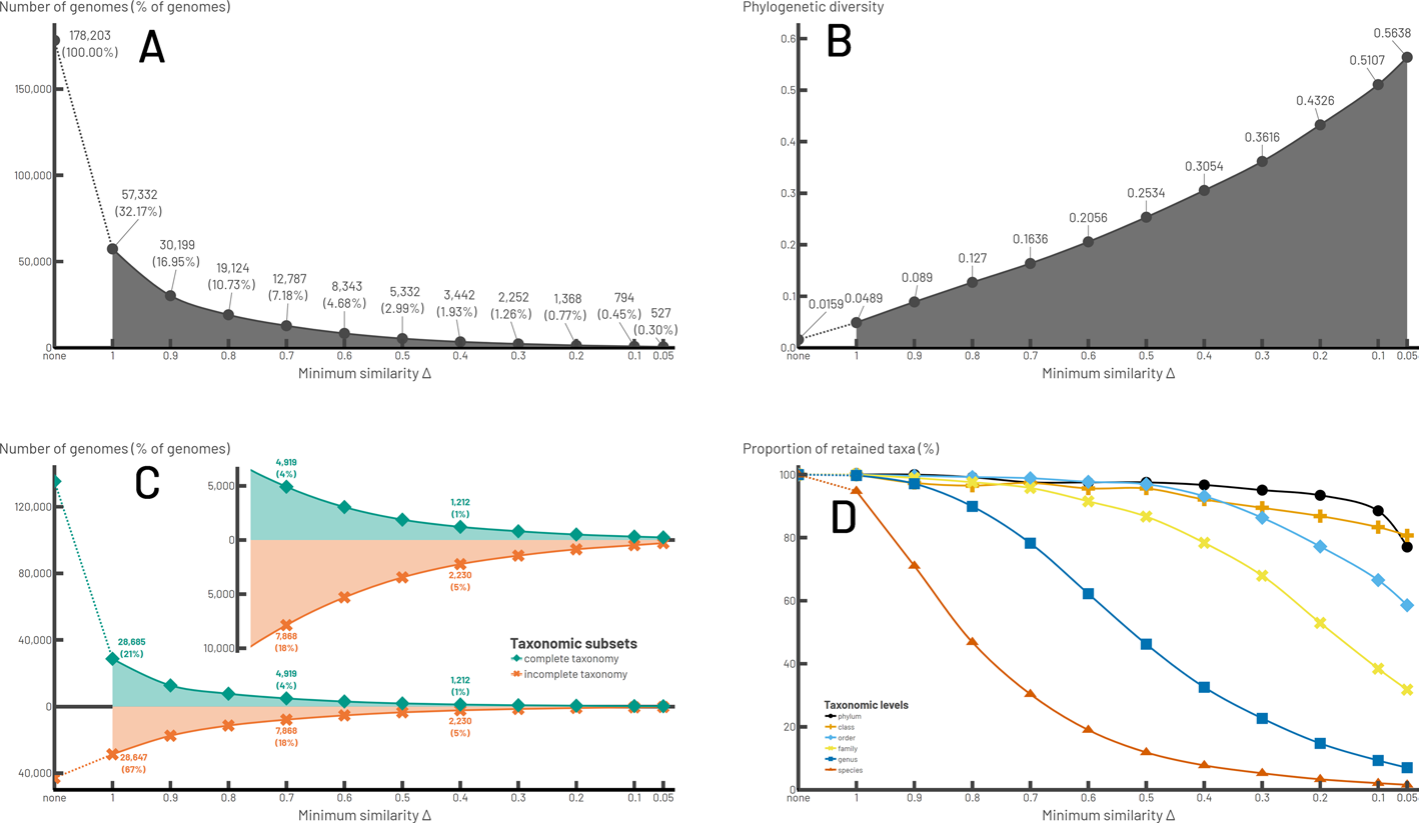
Sampling of the bacterial dataset (178,203 genomes) with MPS-Sampling as a function of Δ. A: Size of the samples. B: Phylogenetic diversity of the samples, computed as the length of all branches of the tree inferred with the sample genomes divided by the number of leaves. C: Detail of the sampling of the 135,315 genomes with a complete taxonomic affiliation and the 42,888 genomes with an incomplete taxonomic affiliation. D: Taxonomic diversity of the samples. The proportion of phyla, classes, orders, families, genera, and species represented in each sample is indicated. For each graph, the solid lines represent a continuous interval of calculable values for Δ, while the dotted lines represent no calculable value of Δ

**Fig. 5:**
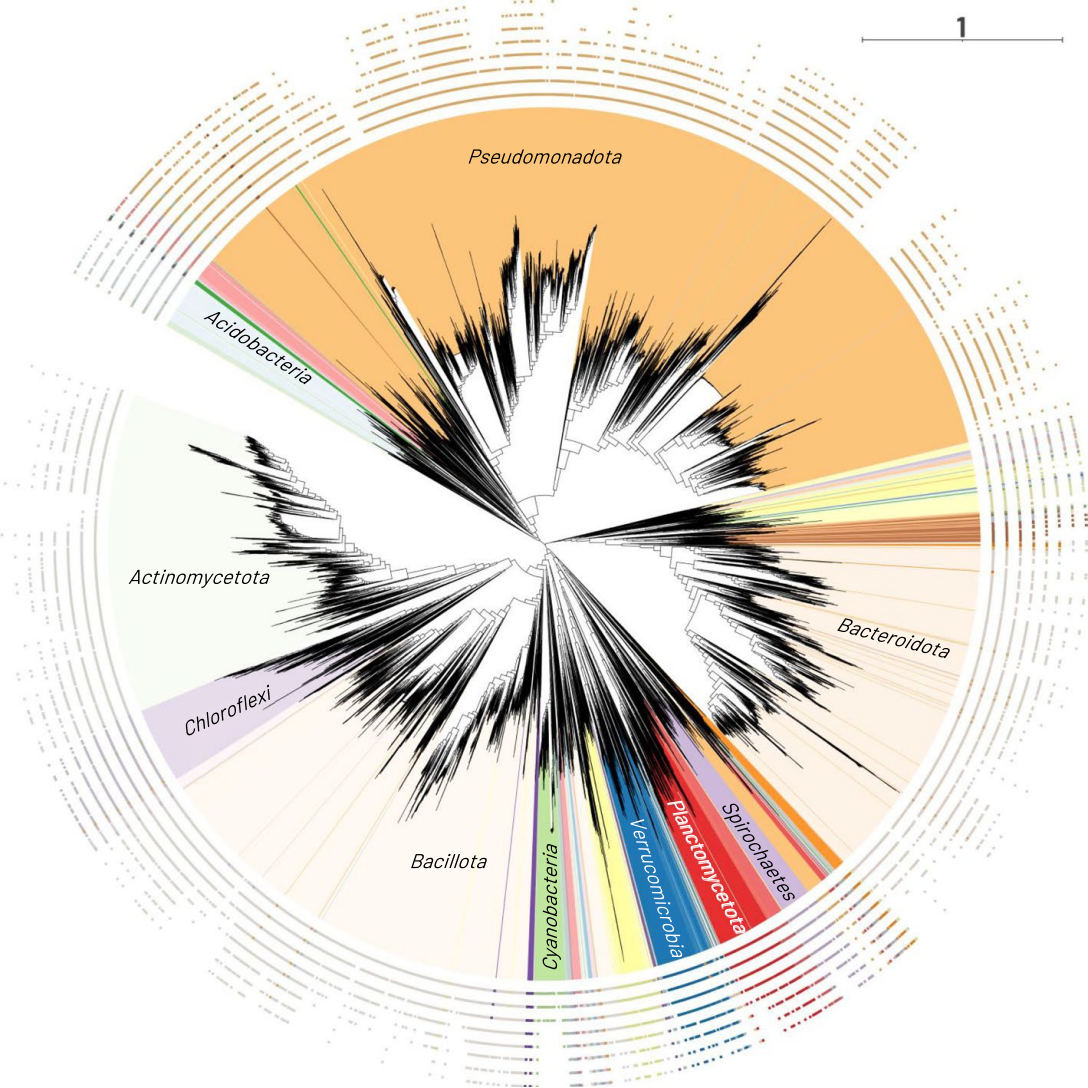
Phylogenetic distribution of the MPS-representatives for *Bacteria*. To make visualization about *Bacteria* possible, a reference bacterial phylogeny of 35,159 genomes has been inferred (Additional file 30). The MPS-representatives from eight samples were mapped on this phylogeny. Each circle represents a sample. Squares correspond to the selected MPS-representatives.  The eight circles correspond, from the innermost to the outermost, to the eight values Δ ∈ {0.7; 0.6; 0.5; 0.4; 0.3; 0.2; 0.1; 0.05}. They contain 12,787, 8,343, 5,332, 3,442, 2,252, 1,368, 794 and 527 MPS-representative genomes, respectively. Samples generated with Δ > 0.7 can not be mapped on this tree because, for these samples, some MPS-representatives are not part of the 35,159 genomes used to build the tree (Additional file 30). The scale bar represents the average number of amino acid substitutions per site in the protein sequences used to infer the tree. The 10 most represented phyla are shown: *Pseudomonadota* (formerly *Proteobacteria*, 12,816 leaves in orange at the top); *Bacillota* (formerly *Firmicutes*, 5,767 leaves in light beige at the bottom); *Bacteroidota* (formerly *Bacteroidetes*, 4,705 leaves in light orange on the right); *Actinomycetota* (formerly *Actinobacteria*, 4,259 leaves in light green on the left); *Chloroflexi* (1,001 leaves in light purple on the left); *Planctomycetota* (formerly *Planctomycetes*, 691 leaves in red below); *Acidobacteria* (615 leaves in light blue top left); *Verrucomicrobia* (560 leaves in dark blue below); *Spirochaetes* (415 purple leaves bottom right); *Cyanobacteria* (413 leaves in green at bottom)

We applied MPS-Sampling to the bacterial dataset (Additional files 26–29). As expected, the lower the value of Δ, the lower the sampling density, with, for example, the selection of 57,332 MPS-representatives (32.17% of the 178,203 genomes) when Δ = 1, 3,474 (1.95%) when Δ = 0.4, and 527 (0.30%) when Δ = 0.05 (Fig. 4A). Mapping the MPS-representatives onto a reference bacterial phylogeny showed that they are well distributed across the tree, even when the number of sampled genomes was very low (0.30%, Δ = 0.05) (Fig. 5 and Additional file 30).

The sampling relevance was assessed by monitoring the phylogenetic and taxonomic diversity of the samples throughout the dereplication process. The phylogenetic diversity increased linearly, from 0.0159 (the whole bacterial dataset) to 0.5638 (Δ = 0.05) (Fig. 4B). This indicates that discarded genomes were indeed phylogenetically the most redundant and that MPS-Sampling is successful in capturing the phylogenetic diversity of the bacterial dataset. A closer look shows that the relative proportion of lineages with incomplete taxonomy increased in the samples (Fig. 4C), which is consistent with the fact they represent a significant part of the bacteria diversity [23].

From a taxonomic point of view, reducing the sampling density led to a progressive reduction in the number of genomes within species (Δ ≤ 1), of species within genera (Δ ≤ 0.9), genera within families (Δ ≤ 0.8), families within orders (Δ ≤ 0.6), and eventually orders and classes within phyla (Δ ≤ 0.4) (Fig. 4D and Additional file 31A). For instance, in the bacterial dataset, species were represented by eight genomes in average. When Δ = 1, around two-thirds of the genomes were eliminated, but almost all the species were kept, each being represented by a single genome in average, meaning that discarded genomes corresponded to redundant genomes within species. Similarly, even when sample sizes were very small (Δ ≤ 0.2), most classes and phyla were conserved, but represented by a few genomes, most often a single genome. Similar trends were observed with the GDTB dataset, except that, in this case, dereplication was more progressive, meaning that for a given Δ, the fraction of MPS-representatives was greater for the GTDB than for the bacterial dataset (Additional file 32). This can be explained by the fact that the GTDB dataset contained twice as many protein families, some of which having faster evolutionary rates than r-prots, which will generate more Lin-clusters and Lin-combinations.

### Comparison of MPS-Sampling, Treemmer, and TaxSampler

**Fig. 6:**
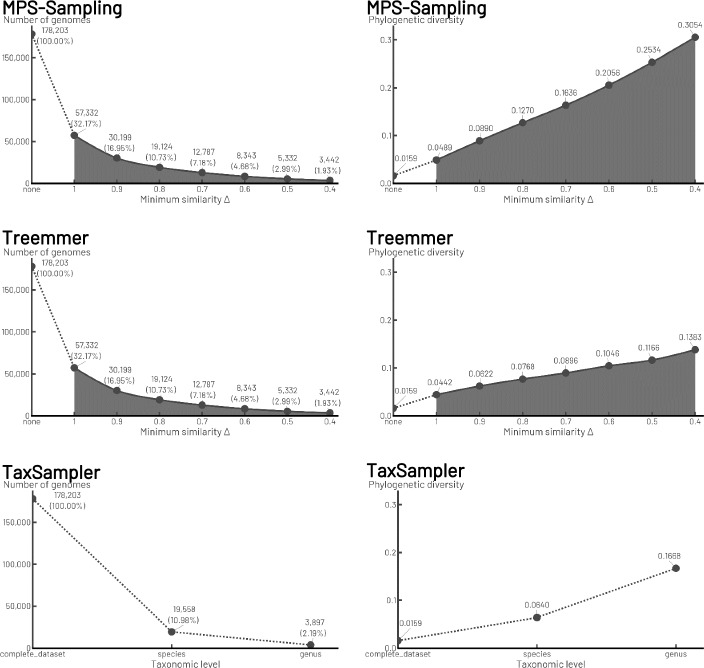
Comparison of sampling rate and phylogenetic diversity for *Bacteria*. Samplings of the bacterial dataset with MPS-Sampling, Treemmer and TaxSampler are compared according to: the sampling rate (left column); the phylogenetic diversity (right column). The solid lines represent the possible values of the parameter Δ with MPS-Sampling or Treemmer, leading to any sample size desired by the user

**Fig. 7:**
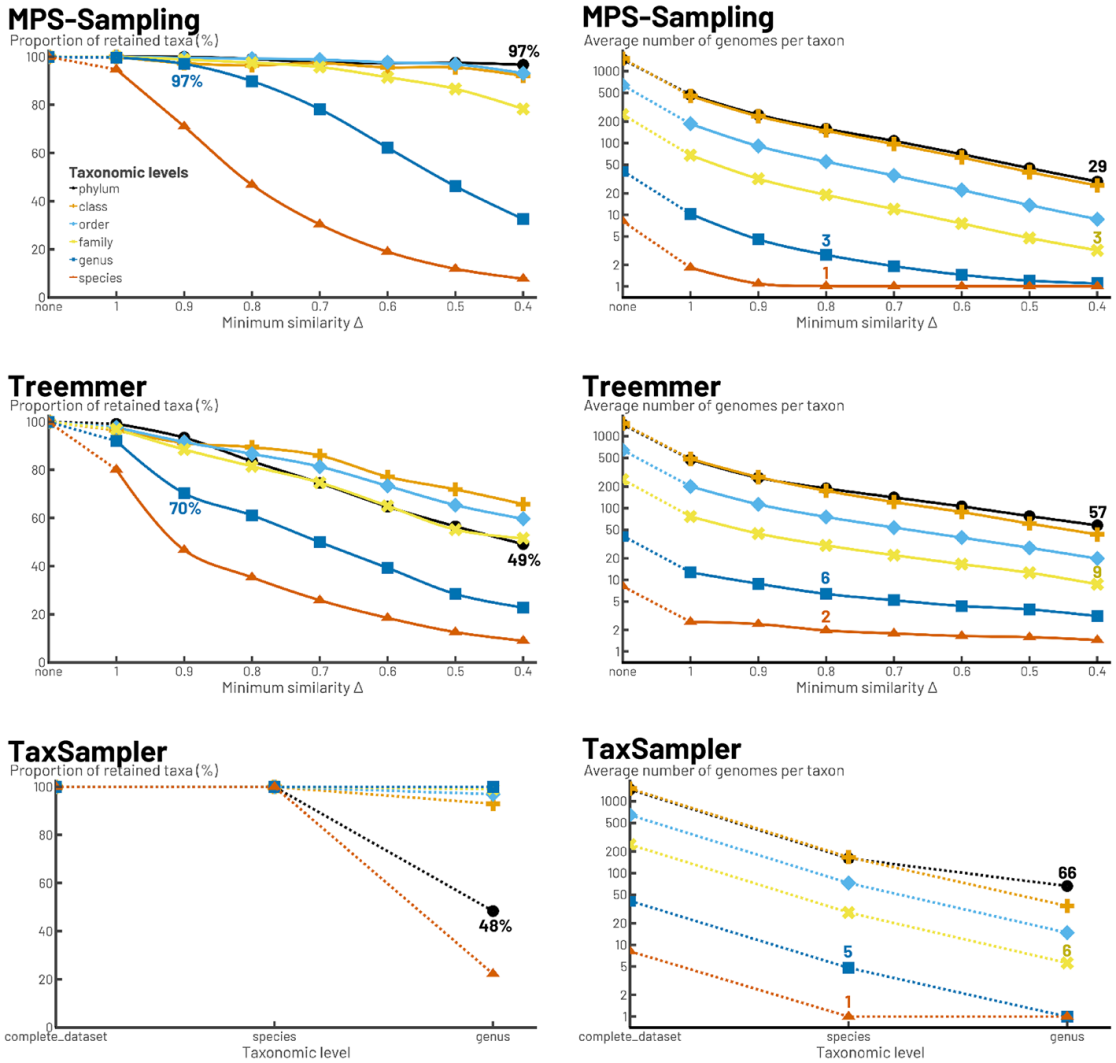
Comparison of taxonomic diversity and taxonomic redundancy for *Bacteria*. Samplings of the bacterial dataset with MPS-Sampling, Treemmer and TaxSampler are compared according to: the taxonomic diversity, with the proportion of retained taxa per level (left column); the taxonomic redundancy, with the average number of genomes per taxon for each level (right column). The solid lines represent the possible values of the parameter Δ with MPS-Sampling or Treemmer, leading to any sample size desired by the user.

We compared the sampling performed by MPS-Sampling, Treemmer and TaxSampler (Fig. 6). MPS-Sampling and Treemmer are able to supply samples of variable size; this is not possible with TaxSampler, which is constrained by the taxonomic levels. For samples of equal size, MPS-Sampling provided samples with higher phylogenetic diversity . For instance, while the phylogenetic diversity of the samples was slightly higher for MPS-Sampling than for Treemmer when Δ = 1 (0.0489 against 0.0442), it was twice as high with MPS-Sampling than with Treemmer when Δ = 0.6 (0.2056 against 0.1046). Regarding TaxSampler (Fig. 6), sampling at the species level led to a sample whose phylogenetic diversity is half that of the sample of similar size provided by MPS-Sampling (i.e. when Δ = 0.4, the phylogenetic diversity of the MPS-sample is 0.1270 against 0.0640). The same held for the sampling at the genus level compared to the sample of similar size provided by MPS-Sampling (i.e. when Δ = 0.8, the phylogenetic diversity of the MPS-sample is 0.3054 compared with 0.1668). In the samples generated by MPS-Sampling, the high-ranking taxa (phyla, classes, orders) are almost all represented from Δ = 1 to Δ = 0.4, whereas in the sample generated by Treemmer with a size corresponding to Δ = 0.4, almost 50% of the phyla are no longer represented. Furthermore, MPS-Sampling is more efficient than both Treemmer and TaxSampler in dereplicating genomes within taxa. For example, also with Δ = 0.4, MPS-Sampling retained two to three times less genomes per family than Treemmer and TaxSampler (3, 9 and 6 genomes kept in average, respectively) (Fig. 7).

**Fig. 8:**
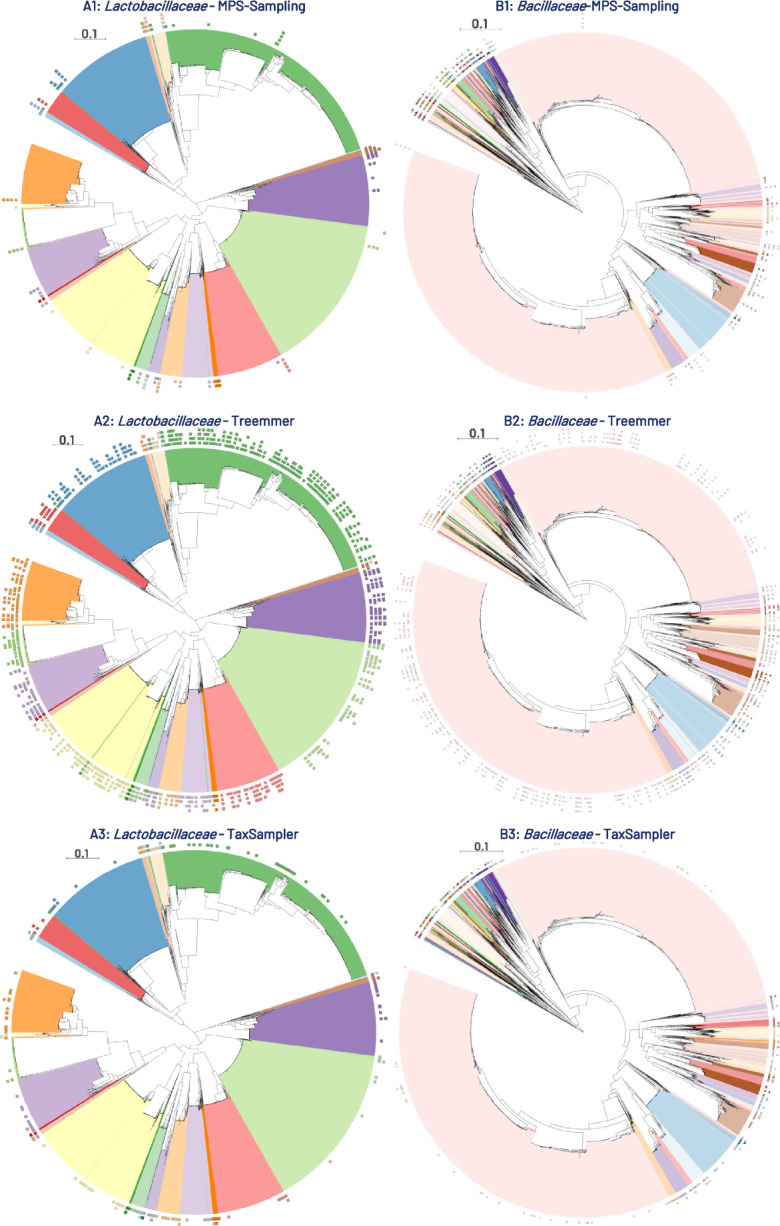
Phylogenetic distribution of MPS-representatives for *Lactobacillaceae* and *Bacillaceae*. Comparison of sampling for two taxonomic families (*Lactobacillaceae* and *Bacillaceae*) by the three methods (MPS-sampling, Treemmer and TaxSampler). The samples are mapped onto reference phylogenies comprising 6,410 genomes (Lactobacillaceae) and 7,113 genomes (Bacillaceae). Each circle represents a sample. Representative genomes are indicated by squares. A: *Lactobacillaceae* - Mapping of the MPS-Sampling, Treemmer, and TaxSampler samples on the phylogeny of the 6,410 genomes of *Lactobacillaceae* contained in the bacterial dataset. A1 - MPS-Sampling: Mapping of the 80, 44, 24 and 12 representative genomes of *Lactobacillaceae* when Δ ∈ {0.7; 0.6; 0.5; 0.4}. A2 - Treemmer: Mapping of the 429, 276, 188 and 124 genomes of *Lactobacillaceae* present in the Treemmer samples of equivalent size to those obtained with MPS-Sampling when Δ ∈ {0.7; 0.6; 0.5; 0.4}. A3 - TaxSampler: Mapping of the representatives of the 394 species and the 33 genera of *Lactobacillaceae* selected by TaxSampler. B - *Bacillaceae*: Mapping of the MPS-Sampling, Treemmer, and TaxSampler samples on the phylogeny of the 7,113 genomes of *Bacillaceae* contained in the bacterial dataset. B1 - MPS-Sampling: Mapping of the 247, 142, 74 and 38 representative genomes of *Bacillaceae* when Δ ∈ {0.7; 0.6; 0.5; 0.4}. B2 - Treemmer: Mapping of the 405, 264, 186 and 114 genomes of *Bacillaceae* present in the Treemmer samples of equivalent size to those obtained with MPS-Sampling when Δ ∈ {0.7; 0.6; 0.5; 0.4}. B3 TaxSampler: Mapping of the representatives of the 693 species and the 108 genera of *Bacillaceae* selected by TaxSampler

To go further, we performed an in-depth analysis of the relevance of the samples produced by MPS-Sampling, Treemmer and TaxSampler for three major bacterial families: the *Lactobacillaceae*, the *Bacillaceae*, and the *Enterobacteriaceae* which presented increasing levels of redundancy (Additional files 33–36). Sampled genomes were mapped on the phylogenies of these families (Fig. 8). The results showed that MPS-sampling provided more reliable samples, unlike Treemmer or TaxSampler, which tended to oversample large taxa with low diversity. As an example, according to its very low phylogenetic diversity, the genus *Lactiplantibacillus* (*Lactobacillaceae*, light green at the bottom-right, Fig. 8A) was correctly reduced to one representative by MPS-Sampling, while it was clearly oversampled by Treemmer (41 to 9 genomes kept) and TaxSampler (18 species kept). As another example, the genus *Bacillus* (*Bacillaceae*), which is composed of 126 closely related species, was clearly oversampled with both TaxSampler and Treemmer (Fig. 8B). More problematic, because *Bacillus* is not monophyletic, sampling at the genus level with TaxSampler will result in the omission of a large part of the *Bacillus* real diversity. The *Enterobacteriaceae* family is another typical example of redundancy, with 99% of the genomes (16,948 out of 17,096) being extremely redundant (see Additional file 35). MPS-Sampling efficiently dereplicated this family, as these redundant genomes represent only 5 to 7% of the *Enterobacteriaceae* genomes sampled by MPS-Sampling. In contrast, Treemmer and TaxSampler dereplicate these redundant genomes less efficiently, as they represent 92 to 94% and 62 to 82% of the sampled *Enterobacteriaceae* genomes, respectively (Additional file 36). These three examples showed that MPS-Sampling succeeded in adapting the sampling density to the level of redundancy in the data and provided samples that are more consistent with the phylogenetic diversity of the data.

## Discussion

MPS-Sampling is a fast, scalable, reliable, sequence similarity-based method for selecting representative sets of genomes. The genome comparison is based on the sequence similarity of a set of protein families (e.g. r-prots, universal single-copy protein families). In this sense, MPS-Sampling is a multilocus sequence analysis (MLSA). Through two steps of genome clustering and matrix calculation, MPS-Sampling is able to process large genomic datasets in an acceptable computational time. MPS-Sampling also includes pre-connection, an optional step, that significantly reduces computation time.

The development of MPS-Sampling was inspired by the work of Sørensen (1948), which uses the Dice index and the hierarchical clustering with complete-linkage [24]. Here, the Dice index is used to calculate the similarity between EGGs. The Dice index has interesting properties, is fast to compute, and has been used in many comparative studies, including for delineating genera boundaries based on conserved proteins [16]. By using hierarchical method with complete-linkage, the intrinsic diversity within each MPS-cluster is controlled: the Dice index between any pair of EGGs is necessarily greater than Δ. MPS-Sampling has therefore a perfect specificity and a very good sensitivity. Indeed, being highly constrained, complete-linkage cannot create false links but can miss some of them, meaning that there is a slight risk of over-sampling, especially for large groups, which was preferred to a possible loss of representativeness. A major issue with hierarchical clustering and complete-linkage is that it does not handle outliers [25]. Here, outliers are singleton genomes that cannot be linked to any other genome and thus correspond to MPS-clusters containing a single genome. It is important to remember that while some of the singletons may be artefactual (e.g. genome sequencing or assembly errors, contaminations), most reflect the real but  undersampled fraction of the biodiversity and/or new lineages.

In this study, MPS-Sampling was used to sample bacterial genomes, but it can be applied to any type of genomes (e.g. complete genomes, metagenome-assembled genomes) form any organisms (e.g. *Archaea*, *Eucarya*, Virus, *Bacteria*), as long as it is possible to assemble families of homologous proteins, since this type of data is used as input. Here, we used r-prots from RiboDB and core protein families from the GTDB as these conserved proteins are well suited for sequence comparison on large evolutionary scales (i.e. from species to phyla) [21, 26, 27]. This has been confirmed by the reliability of MPS-Sampling at both large and small evolutionary scales (*Bacteria*, *Lactobacillaceae*, *Bacillaceae*, *Enterobacteriaceae*). However, at smaller evolutionary scales (e.g., intra-species), using nucleotide sequences or faster evolving protein families would be more appropriate.

MPS-Sampling is sensitive to the quality of the data, as are all algorithms. However, MPS-Sampling is relatively robust to protein family assembly errors and missing data (e.g. gene loss, incomplete genome sequencing) because it encapsulates information from many protein families. Indeed, let's call F a protein family that is present in one set of genomes (called set A) but missing in another set of genomes (called set B). In this case, the sequences of F are considered when comparing genomes from set A, but the F family is ignored when comparing genomes from set B (Fig. 2 and Additional file 8). When comparing genomes from set A with those from set B, the F family is considered and will help to discriminate them. As a result, MPS-Sampling uses present sequences when they are present, ignores them when they are absent, and tends to separate genomes with different protein family sets. In the case of rprots, even after filtering out incomplete data, the dataset contains 2.78% missing sequences. In this case, MPS sampling works well. However, the more missing sequences the data contains, the less relevant the analysis becomes. If we take this line of reasoning to its logical conclusion, would it make sense to compare two proteomes that do not share any homologous sequences?

Consequently, MPS-Sampling can be used with non-core protein families. MPS-Sampling is also relatively resistant to horizontal gene transfer (HGT), genome chimerism, and protein family assembly errors because the signal carried by the xenologous and non-homologous sequences will be dominated by the signal carried by the other proteins, allowing the corresponding genomes to be correctly linked. However, high levels of HGT or systematic error in protein family assembly can affect the sampling procedure. For example, if more than 30% of the proteins of genome A from taxon T have been acquired by HGT from a single donor, genome A will be correctively grouped with other genomes of T when Δ ≤ 0.7, but not when Δ ≥ 0.7. In this case, the genome A will be isolated from the other genomes to form a singleton MPS-cluster.

## Conclusions

MPS-Sampling is a new method for selecting samples of representative genomes from a huge database, based on Multi-Proteins Similarity (MPS). Our study shows that MPS-Sampling was particularly performant to dereplicate a large dataset of bacterial genomes, holding most of the evolutive diversity of the original set, both taxonomically and phylogenetically, and at various evolutionary scales. MPS-Sampling is still consistent when taxonomy is misleading and diverging from phylogeny.

## Supplementary Information

Additional file 1

## Data Availability

The datasets used in this study are available in the figshare repository, https://figshare.com/articles/dataset/Article_-_MPS-Sampling/24552160.

## Abbreviations

EGG: Elementary group of genomes, the first clustering of genomes realized by MPS-Sampling during the step 2
eValue: The parameter of Linclust concerning the e-value
minCov: The parameter of Linclust concerning the minimal coverage
MPS: Multi-proteins similarity
MPS-cluster: The second and last clustering of genomes realized by MPS-Sampling during the step 4
minSeqID: The parameter of Linclust concerning the minimal sequence identity
r-prot: Ribosomal protein
