# Supplementary material for: Multi-proteins similarity-based sampling to select representative genomes from large databases
Source: BMC Bioinformatics. 2025 May 6;26:121. doi: 10.1186/s12859-025-06095-3 (PMC12057276; doi:10.1186/s12859-025-06095-3)
Supplement: Supplementary file 1 — Additional file 1 [file 12859_2025_6095_MOESM1_ESM.pdf]

Additional files

Multi-Proteins Similarity-based sampling to select representative genomes from large databases

Rémi-Vinh Coudert[1,2,*], Jean-Philippe Charrier[2], Frédéric Jauffrit[2], Jean-Pierre Flandrois[1,*], Céline Brochier-Armanet[1,3,*]

[1] Universite Claude Bernard Lyon 1, LBBE, UMR 5558, CNRS, VAS, Villeurbanne, F-69622, France

[2] Microbiology Research & Development, bioMérieux SA, 376 Chemin de l'Orme, 69280 Marcy-l'Étoile, France

[3] Institut Universitaire de France

*To whom correspondence should be addressed.

List of Additional Files

Additional file 1 – Growth of available genomic data over time 3
Additional file 2 – Unbalanced representativity in available genomic data 4
Additional file 3 – Available sets of representative genomes 5
Additional file 4 – Technical encoding 6
Additional file 5 – Flowchart of MPS-Sampling 8
Additional file 6 – Entity relationship diagram (ERD) of MPS-Sampling 9
Additional file 7 – From Lin-combinations to pre-connection 10
Additional file 8 – Dice index and similarity matrix 11
Additional file 9 – Calculation of the similarity matrix 12
Additional file 10 – Priority rules for the choice of the MPS-representative genomes 15
Additional file 11 – Centrality criterium 16
Additional file 12 – Preparation of the bacterial dataset 19
Additional file 13 – Generation of the artificial bacterial dataset 20
Additional file 14 - Choice of the parameters of MPS-Sampling (without pre-connection) 21
Additional file 15 – Median number of Lin-clusters according to minimum sequence identity 22
Additional file 16 – Median number of Lin-clusters according to coverage mode 23
Additional file 17 – Median number of Lin-clusters according to eValue 24
Additional file 18 – Median number of Lin-clusters according to minimum coverage 25
Additional file 19 – Choice of the parameters of MPS-Sampling (for pre-connection) 26
Additional file 20 – Size of the largest pre-connected component depending on MinNbLinclusters 27
Additional file 21 – Number of pre-connected components depending on MinNbLinclusters 27
Additional file 22 - Phylogenetic reconstruction 28
Additional file 23 – TaxSampler, a homemade-software for sampling genomes based on taxonomy 29
Additional file 24 – Computational time MPS-Sampling concerning the 178,203 genomes of the bacterial dataset 30
Additional file 25 – Computational time 31
Additional file 26 – Intermediate results of MPS-Sampling concerning the bacterial dataset 33
Additional file 27 – Number of MPS-clusters where each selection rule was applied 35
Additional file 28 – Taxonomic statistics about each investigated subset 36
Additional file 29 – Phylogenic statistics about each phylogenetic inference 36
Additional file 30 – Construction of a reference bacterial phylogeny 38
Additional file 31 – Monitoring the dereplication process of the bacterial dataset 40
Additional file 32 – Monitoring the dereplication process of the GTDB dataset 41
Additional file 33 – Sampling of *Lactobacillaceae*, *Bacillaceae* and *Enterobacteriaceae* 42
Additional file 34 – Reduction of three taxonomic families 44
Additional file 35 – Phylogenetic mapping of MPS-representatives for three bacterial families 45
Additional file 36 – Phylogenetic mapping for *Enterobacteriaceae* 46

Additional file 1 – Growth of available genomic data over time

From the NCBI website, the following file was downloaded: ftp://ftp.ncbi.nlm.nih.gov/genomes/GENOME_REPORTS/prokaryotes.txt.

It represents the available genomic data of GenBank. All rows until and including 2022 were considered. A cumulated count by year was computed and showed in the bar plot below. Among them, the draft genomes (i.e. scaffolds and contigs) were colored in light blue at the top of each bar and complete genomes in dark blue.

The cumulated amount doubled every 2 or 3 years and reached 491,300 genomes at the end of 2022. At this time, only 8% of the data were complete genomes while the remaining 92% were only draft genomes.

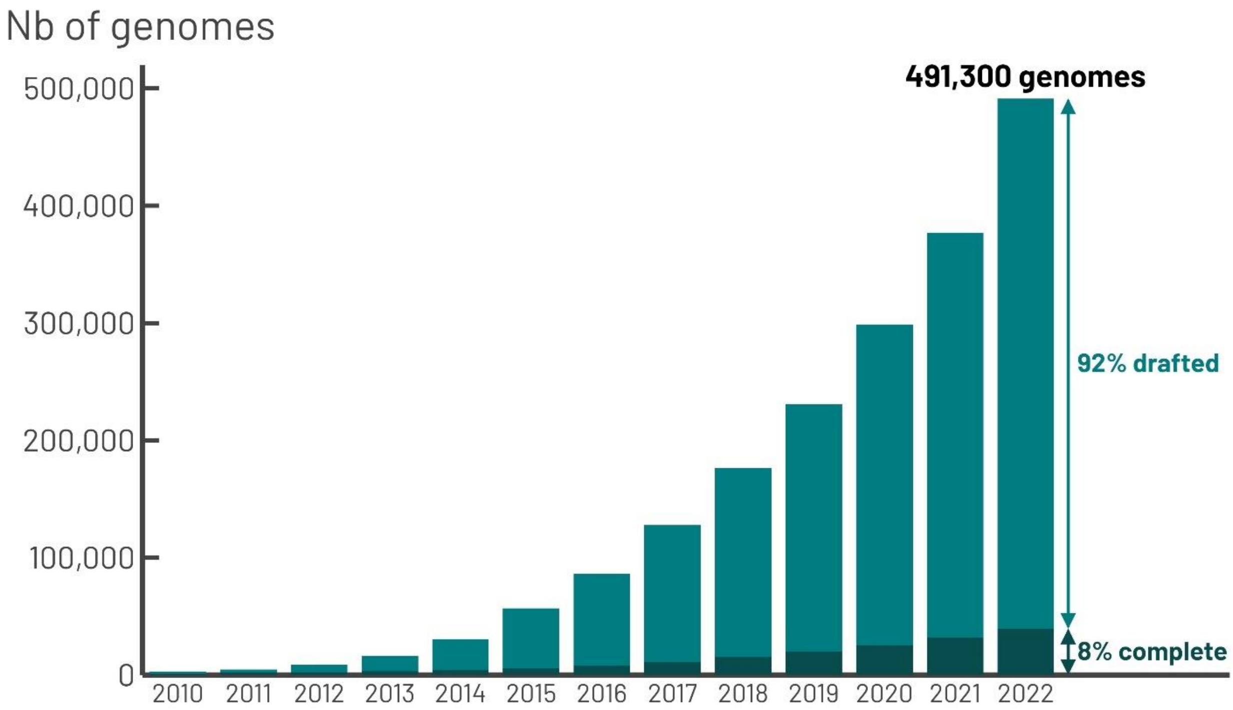

Additional file 2 – Unbalanced representativity in available genomic data

From the NCBI website, the following file was downloaded: ftp://ftp.ncbi.nlm.nih.gov/genomes/GENOME_REPORTS/prokaryotes.txt.

It represents the available genomic data of GenBank. All rows until and including 2022 were considered. Genomes whose species name ending with sp., bacterium, or archaeon, were classified as "undescribed". Remaining genomes were classified as "described". Among them, the genomes of the 10 most represented species were highlighted, as mentioned in the legend (*Escherichia coli*, *Staphylococcus aureus*...).

In 2022, 38% of the genomes correspond to "undescribed" genomes and 62% to described genomes, among which 43% represent only 10 species, the most sequenced ones.

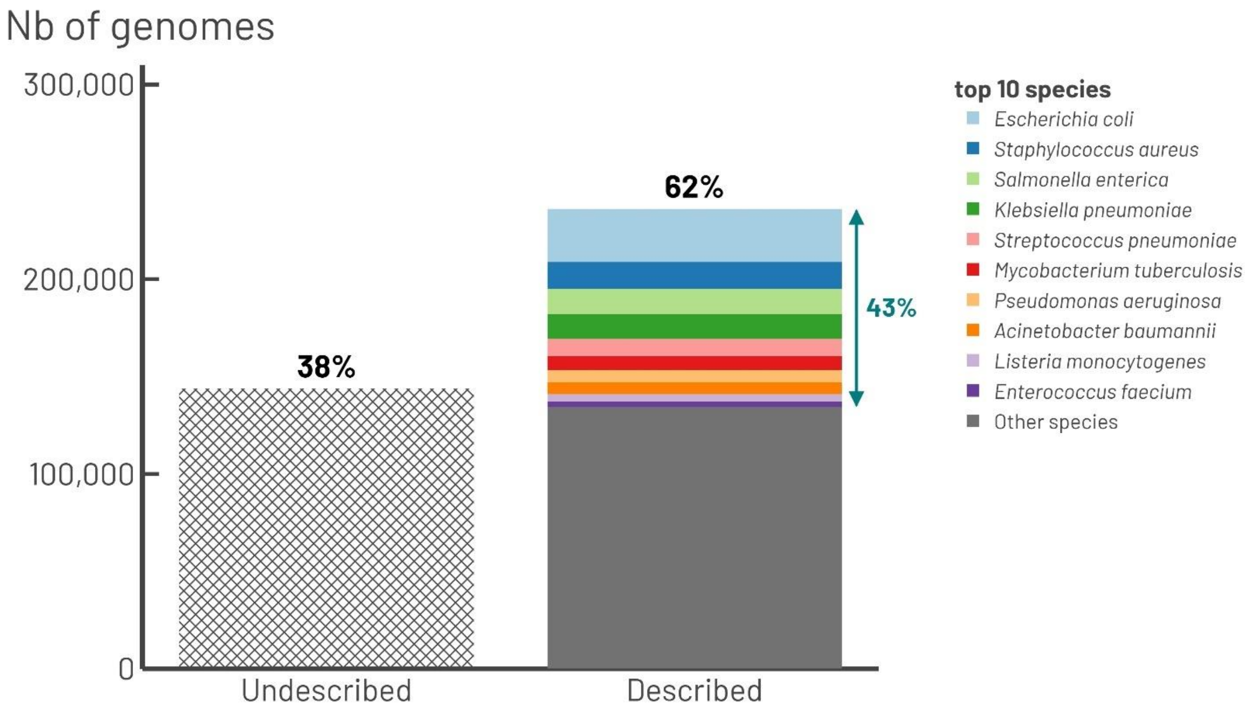

Additional file 3 – Available sets of representative genomes

Several consortia provide ready-to-use sets of representative genomes. For instance of 2023/01/27, the NCBI RefSeq database [1] references 17,145 representative genomes (including 16,557 of Bacteria), Uniprot [2] 22,121 proteomes (including 8,821 for *Bacteria*), and Ensembl! [3] 33,316 genomes (including 31,332 for *Bacteria*) respectively. RefSeq-representative genomes are " computationally or manually selected as a representative from among the best genomes available for a species or clade". They are "chosen among eligible assemblies based on [some] criteria", the first criterium being the "manual selection". The UniProt reference proteomes are "selected among all proteomes (manually and algorithmically, according to a number of criteria) to provide broad coverage of the tree of life". Ensembl-representative genomes [3] are chosen among UniProt according to automatic dereplication rules. These libraries bring together a qualitative and supposedly representative sampling of the taxonomic diversity of genomic data. These three reference sets of genomes include proteomes of interest for biomedical and biotechnological research, and are thus highly impacted by socio-induced biases. The advantage for the users is that they do not have to manage the data sampling step. However, the use of these libraries also has limitations. First, the users have no control over the selection of genomes, so some taxonomic groups of interest for the user may not be represented. Second, the sampling density and the redundancy of the data are not controlled, which may require a second sampling step. Finally, most of the time, these libraries do not include the genomes of undescribed organisms.

Additional file 4 – Technical encoding

Distribution

MPS-Sampling is distributed as a Snakemake pipeline available at:
https://github.com/rvcoudert/MPS_Sampling

Snakemake is a scalable, Python-based workflow manager [4]. The execution of a Snakemake pipeline is managed by a master script, called a SnakeFile and coded in Python, which constructs a master diagram and lists the tasks to be performed. The master script automatically calls secondary scripts to accomplish the necessary tasks. The full MPS-Sampling master diagram is detailed in a flowchart shown in the Additional file 5. All data tables used during the MPS-Sampling workflow are detailed in an entity relationship diagram (ERD) in Additional file 6. For MPS-Sampling, it is chosen to manage computing environments of different scripts using Conda [5]. Conda is an environment manager that automatically installs all necessary dependencies and prepares the appropriate environment when running a secondary script with Snakemake. For MPS-Sampling, the main dependency is the Linclust package [6] from the MMseqs2 suite [7]. All other intermediate scripts use the R language. For MPS-Sampling, there are therefore two Conda environments used: one to launch Linclust and one to launch the R scripts.

Format of input files

MPS-Sampling has two types of input files: FASTA files for protein sequences and a CSV (Comma-Separated Values) file for indexing genomes.

There is one FASTA file per protein family. In each FASTA file, the sequences must be named after the genome to which they belong: the name of the sequences is the primary key linking sequence and genome. For example, in **Erreur ! Source du renvoi introuvable.**, the $\mathbf{g_B}$ genome has a sequence for the **u L1**, **uL3** and **uL4** families, but not for the **uL2** family. This means that the FASTA files corresponding to the **ul1**, **uL3** and **uL4** families each contain a single-copy sequence belonging to the $\mathbf{g_B}$ genome. On the other hand, the FASTA file corresponding to the **uL2** family does not contain any $\mathbf{g_B}$ genome sequence.

The CSV file constituting the genome index has two columns separated by a comma: **genomeAccession** and **priority_score**.

- **genomeAccession**.
- **priority_score** defines the order of priority for the selection of MPS-representative genomes. The higher a genome score, the more priority it is chosen. This score constitutes the first priority rule when choosing representative MPS-genomes (Additional file 10).

Format of intermediate files

The intermediate files are described in the entity relationship diagram (ERD) in Additional file 6. All files are of CSV type, except the TSV files generated by Linclust and two RDS files to respectively export the similarity matrix and hierarchical clustering. TSV files are simply CSV files but with a tab as separator instead of a comma (Tabulation-Separated Values). The RDS format is chosen to export the similarity matrix and hierarchical clustering because of its practicality. It allows to quickly write and read data with R as well as compress backups.

Format of output files

The list of the selected MPS-representative genomes is accessible in the table **MPS-representatives** containing only a single column: **MPS-representative** the accession number of the MPS-representative genomes. The link between the input genomes and the MPS-representative genomes is accessible in the table **MPS-links**.

Running MPS-Sampling

An example to perform several runs of MPS-Sampling in a single-line command is :

```
snakemake --use-conda --cores 10 -s /home/MPS-Sampling/Snakefile -d /home/Data/RiboDB -config deltas=[0.5,1]
```

This command line launches MPS-Sampling in parallelization with 10 cores, using the SnakeFile localized in "/home/MPS-Sampling/Snakefile", applied to the data set localized in "/home/Data/RiboDB", in order to generate two samples with $\Delta = 0.5$ and $\Delta = 1$.

Additional file 5 – Flowchart of MPS-Sampling

This flowchart shows the data analysis processed by MPS-Sampling, centralized thanks to a Snakemake pipeline. Files are colored in light green. The only external program is Linclust and colored in black. All other tasks are carried out by home-made R scripts, colored in strong blue.

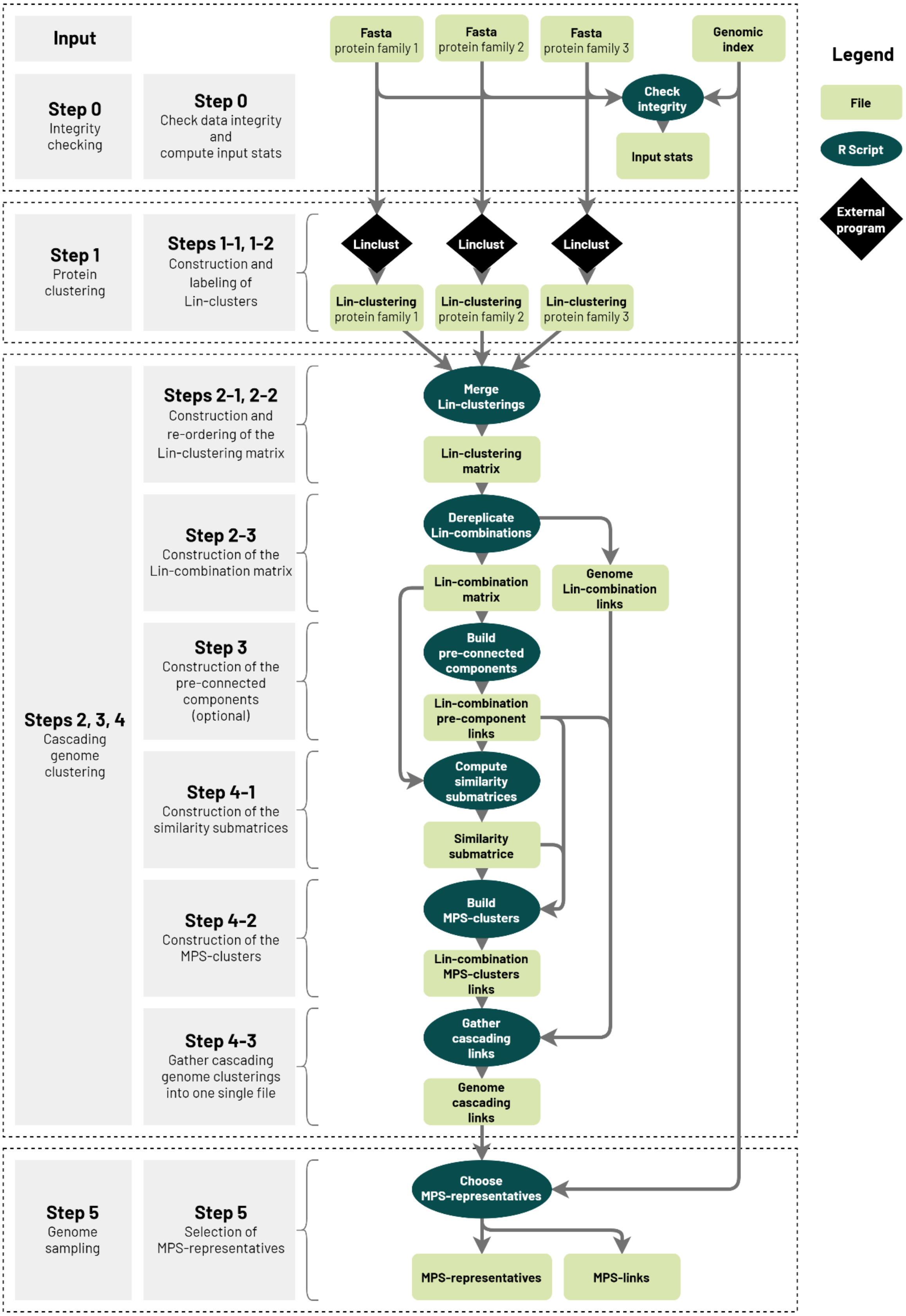

Additional file 6 – Entity relationship diagram (ERD) of MPS-Sampling

This entity relationship diagram (ERD) [8] below details the relational schema of MPS-Sampling.

Each box presents a distinct data table with its columns. Primary keys are shaded in grey and links between columns are indicated by arrows.

For example, the **Genome index** table has three columns: (i) **genome accession**, which represents the accession number of the genome and is the primary key of the table; (ii) **priority score**, which represents the priority score for choosing MPS-representative genomes (Additional file 10); and (iii) **genome hash**, which contains the hashing value of the accession number of the genome, to separate equivalent choices using a pseudo-random criterion (Additional file 10). The primary key genome accession is shared with the tables Fasta files and Genome Lin-combination links. The type of arrows indicates that there is strictly one entry in the Genome index table for one entry in the Genome Lin-combination links table. This is consistent because each genome belongs strictly to one and only one Lin-combination. On the other hand, for each entry in Genomic index, there is zero or one entry in the Fasta files. This is still consistent because the absence of a sequence in a genome is authorized (zero entry), as is a single-copy sequence (one entry), but duplicated sequences are not accepted (multiple entries forbidden). Conversely, each sequence must have one name and one name only, so each sequence is attached to strictly one genome.

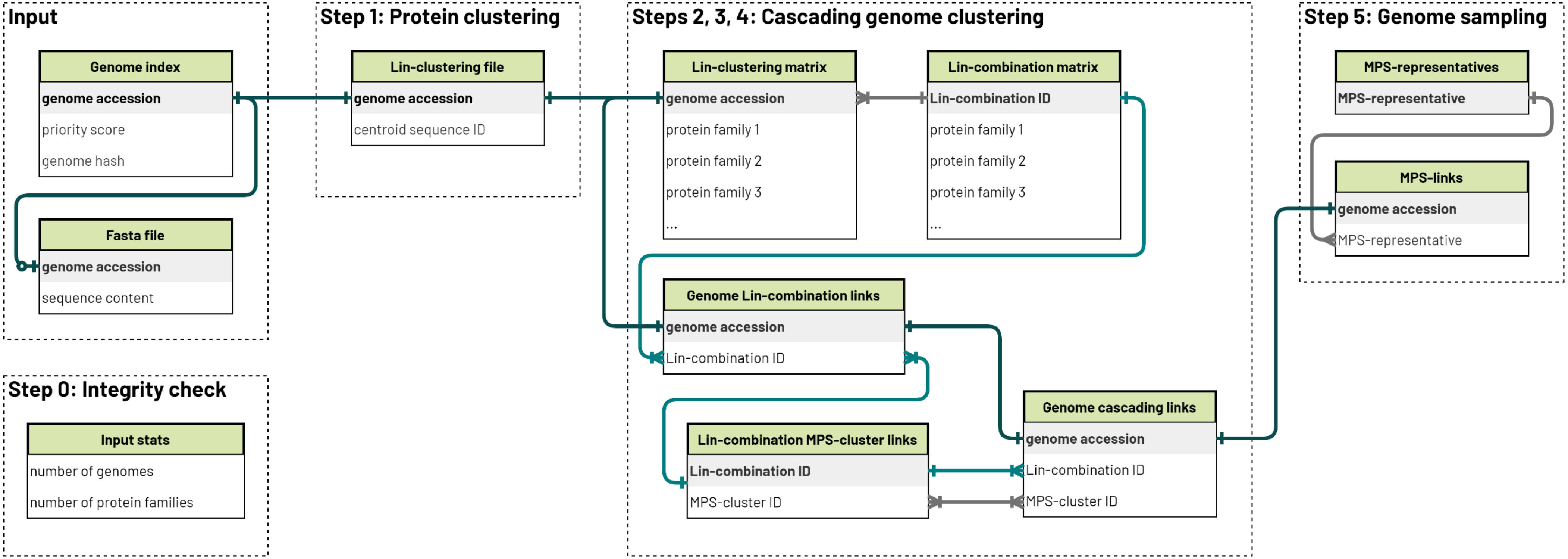

Additional file 7 – From Lin-combinations to pre-connection

Example of the construction of a pre-connected component during the pre-connection, with the parameter minNbLinclusters = 2. From a starting Lin-combination, close Lin-combinations are iteratively absorbed according to a single common reference of target Lin-clusters.

- **Initialization:** The pre-connected component is initialized with a given Lin-combination: **Comb1**.
- **Iteration 1:** Lin-combinations sharing at least 2 Lin-clusters are searched. Here, 2 Lin-combinations, **Comb2** and **Comb3**, share at least 2 Lin-clusters with **Comb1**. These 2 Lin-combinations are added to the pre-connected component. This is the aggregative aspect of pre-connection. For the next iteration, the list of Lin-clusters to be searched is updated with the information carried by **Comb2** and **Comb3**.
- **Iteration 2:** According to the updated list of Lin-clusters, **Comb4** is identified. This Lin-combination is added, and the current pre-connected component contains now 4 Lin-combinations: **Comb1**, **Comb2**, **Comb3**, and **Comb4**. The list of Lin-clusters to be searched is again updated.
- **Iteration 3:** No compatible Lin-combination is found. Thus the pre-connected component is now stable, and its delineation ends.

To resume, from the Lin-combination **Comb1**, a pre-connected component has been delineated through 3 iterations. It contains 4 Lin-combinations: **Comb1**, **Comb2**, **Comb3**, and **Comb4**. The process could be used to build a second pre-connected component. Starting from the Lin-combination **Comb5**, it ends containing three Lin-combinations: **Comb5**, **Comb6**, and **Comb7**.

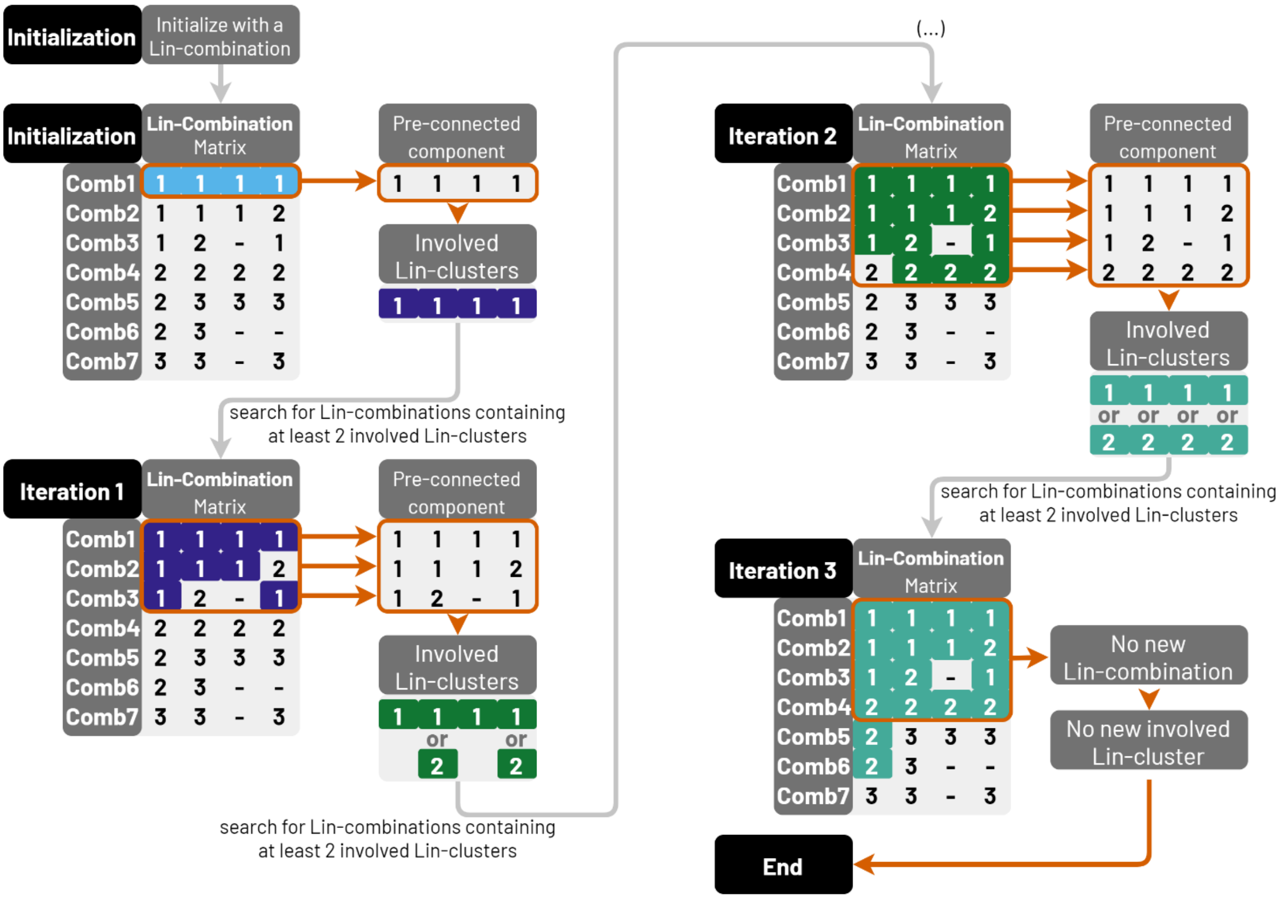

Additional file 8 – Dice index and similarity matrix

Noting A and B the two compared Lin-combinations, the Dice index is given by the formula:

$$\frac{2 \times card(A \cap B)}{card(A) + card(B)}$$

The figure below illustrates the computation of the Dice index between **Comb6** (2, 3, –, -) and **Comb7** (3, 3, –, 3). The number of common Lin-clusters and the total number of Lin-clusters (without missing values) are respectively 2 and 5. The Dice index is thus equals to 2 / 5.

The Dice index handles missing values. It completely ignores the missing values when the protein family is missing in both Lin-combinations (e.g. protein family 3, missing in **Comb6** and **Comb7**) and takes into account when a protein is missing in only one Lin-combination (e.g. protein family 4, missing in **Comb6** but present in **Comb7**).

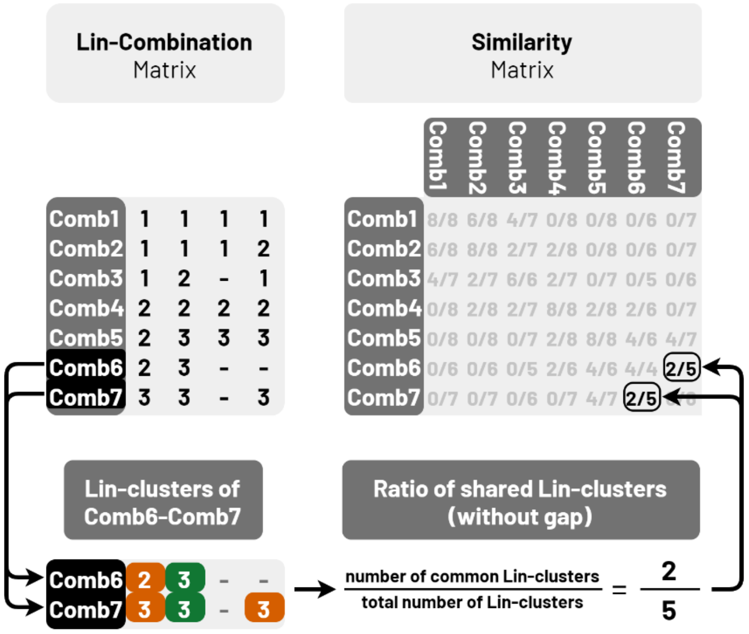

If the pre-connection is used, only the square submatrices corresponding to the pre-connected components are computed. In the figure below, two submatrices have been computed, corresponding to the two pre-connected components delineated in the Additional file 7. Five MPS-clusters have been built, corresponding to the five MPS-clusters present in the **Erreur ! Source du renvoi introuvable.**.

Additional file 9 – Calculation of the similarity matrix

To minimize quadratic complexity, the similarity matrix is calculated per column using the properties of the Dice index (Additional file 8). For two sets A and B, the formula of the Dice index is:

$$\frac{2 \times \boldsymbol{card}(\boldsymbol{A} \cap \boldsymbol{B})}{\boldsymbol{card}(\boldsymbol{A}) + \boldsymbol{card}(\boldsymbol{B})}$$

Let $\boldsymbol{B}$ be a given Lin-combination, calculating the column corresponding to $\boldsymbol{B}$ is equivalent to calculating the Dice index between $\boldsymbol{B}$ and all the $\boldsymbol{N}$ other Lin-combinations $\{\boldsymbol{A_i}\}_{i=1}^{N}$ ($\boldsymbol{B}$ included), i.e. the vector of size $\boldsymbol{N}$:

$$\left(\frac{2 \times \boldsymbol{card}(\boldsymbol{A_i} \cap \boldsymbol{B})}{\boldsymbol{card}(\boldsymbol{A_i}) + \boldsymbol{card}(\boldsymbol{B})}\right)_{i=1}^{N} = \frac{2 \times (\boldsymbol{card}(\boldsymbol{A_i} \cap \boldsymbol{B}))_{i=1}^{N}}{(\boldsymbol{card}(\boldsymbol{A_i}))_{i=1}^{N} + \boldsymbol{card}(\boldsymbol{B})}$$

The idea is to calculate the numerator-vector and the denominator-vector separately. In the numerator-vector, the main factor is $(\boldsymbol{card}(\boldsymbol{A_i} \cap \boldsymbol{B}))_{i=1}^{N}$, which simply is the number of Lin-clusters in common between $\boldsymbol{B}$ and the other Lin-combinations $\{\boldsymbol{A_i}\}_{i=1}^{N}$. In the denominator-vector, the main term is $(\boldsymbol{card}(\boldsymbol{A_i}))_{i=1}^{N}$, which is just the number of Lin-clusters per Lin-combination, plus the number of Lin-clusters from $\boldsymbol{B}$.

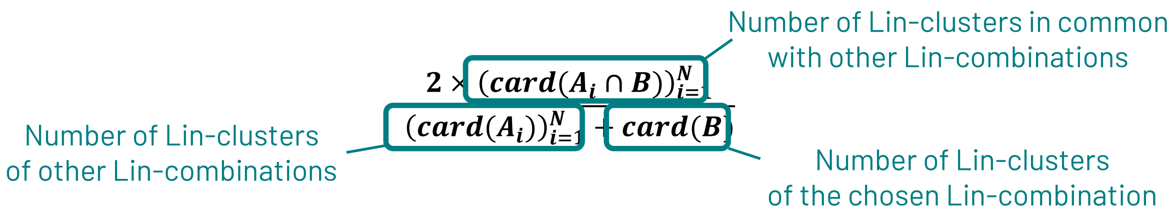

This allows the calculation of the numerator-vector and denominator-vector separately. The figure below presents the computation of the Dice indexes between **Comb3** and the 7 other Lin-combinations.

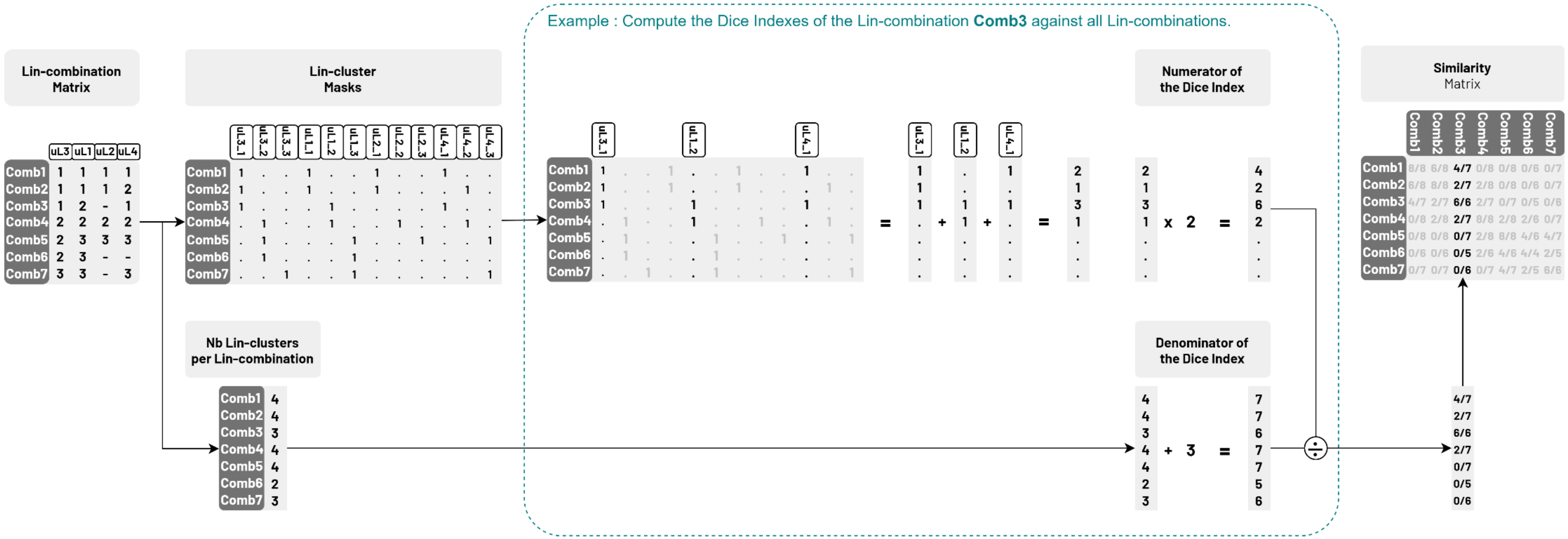

Firstly, the Lin-combination matrix is transformed through one-hot encoding [9]. This means calculating the mask for each Lin-cluster. The mask of a Lin-cluster is a binary vector over all the protein sequences evaluating membership of the Lin-cluster: 1 if the sequence belongs to the Lin-cluster, 0 otherwise. The resulting matrix is called the mask matrix. This matrix has as many columns as there are different Lin-clusters among all the protein families and as many rows as there are Lin-combinations. The mask matrix has 12 columns because there are 12 different Lin-clusters among the 4 protein families considered; it also has 7 rows because there are 7 different Lin-combinations. At the same time, the number of Lin-clusters per Lin-combination is calculated and stored in a vector.

The numerator-vector is calculated. The masks of the Lin-clusters in the concerned Lin-combination are added together to obtain the number of Lin-clusters in common with all the other Lin-combinations. Here, **Comb3** has 3 Lin-clusters: **ul3_1**, **ul1_2** and **ul4_1**. The masks of these 3 Lin-clusters are added together to obtain the number of Lin-clusters in common between **Comb3** and all the other Lin-combinations:

$$v = \begin{pmatrix} 1 \\ 1 \\ 1 \\ . \\ . \\ . \\ . \end{pmatrix} + \begin{pmatrix} . \\ . \\ 1 \\ 1 \\ . \\ . \\ . \end{pmatrix} + \begin{pmatrix} 1 \\ . \\ 1 \\ . \\ . \\ . \\ . \end{pmatrix} = \begin{pmatrix} 2 \\ 1 \\ 3 \\ 1 \\ 0 \\ 0 \\ 0 \end{pmatrix}$$

The digit **2** in the first coordinate means that **Comb3** shares 2 *Lin-clusters* with **Comb1**. This vector $\boldsymbol{v}$ is multiplied by 2 to obtain the numerator-vector of the Dice indices. For **Comb3**, the numerator-vector obtained is:

$$\mathbf{2} \times \boldsymbol{v} = \mathbf{2} \times \begin{pmatrix} \mathbf{2} \\ \mathbf{1} \\ \mathbf{3} \\ \mathbf{1} \\ \mathbf{0} \\ \mathbf{0} \\ \mathbf{0} \end{pmatrix} = \begin{pmatrix} \mathbf{4} \\ \mathbf{2} \\ \mathbf{6} \\ \mathbf{2} \\ \mathbf{0} \\ \mathbf{0} \\ \mathbf{0} \end{pmatrix}$$

The denominator-vector is then calculated. The number of Lin-clusters per Lin-combination is added to the number of Lin-clusters in the concerned Lin-combination. **Comb3** has 3 Lin-clusters, so the obtained denominator-vector is:

$$\begin{pmatrix} \mathbf{4} \\ \mathbf{4} \\ \mathbf{3} \\ \mathbf{4} \\ \mathbf{4} \\ \mathbf{2} \\ \mathbf{3} \end{pmatrix} + \mathbf{3} = \begin{pmatrix} \mathbf{7} \\ \mathbf{7} \\ \mathbf{6} \\ \mathbf{7} \\ \mathbf{7} \\ \mathbf{5} \\ \mathbf{6} \end{pmatrix}$$

By dividing the numerator-vector by the denominator-vector, the column of the similarity matrix corresponding to the Lin-combination **Comb3** is obtained, i.e.:

$$\begin{pmatrix} \mathbf{4} \\ \mathbf{2} \\ \mathbf{6} \\ \mathbf{2} \\ \mathbf{0} \\ \mathbf{0} \\ \mathbf{0} \end{pmatrix} / \begin{pmatrix} \mathbf{7} \\ \mathbf{7} \\ \mathbf{6} \\ \mathbf{7} \\ \mathbf{7} \\ \mathbf{5} \\ \mathbf{6} \end{pmatrix} = \begin{pmatrix} \mathbf{4/7} \\ \mathbf{2/7} \\ \mathbf{6/6} \\ \mathbf{2/7} \\ \mathbf{0/7} \\ \mathbf{0/5} \\ \mathbf{0/6} \end{pmatrix}$$

Calculating the similarity matrix by column saves time compared to calculating it cell by cell and thus limits the quadratic complexity.

Additional file 10 – Priority rules for the choice of the MPS-representative genomes

The MPS-representative genomes are selected according to rigorous priority rules, which favor:

(1) The genomes with the highest user-defined priority score. For our analysis, a priority score is given to each genome based on several priority criteria. For example, a genome indicated RefSeq-representative and completed **(RC)** has a score of 32 + 8 = 40.

(2) The genomes with the largest distribution across the protein families used as input, i.e. the genomes with the fewest missing protein families.

(3) The genomes with the best centrality within the MPS-cluster considered, i.e. with the highest possible average Dice index to the other genomes. This is equivalent to the genomes whose Lin-clusters are the most frequent (Additional file 11).

(4) Genomes whose name has the lowest hash value. Hash values are calculated with the function **hash( )** of the package **R rlang**.

Step			Description
**1**	**Tag**	**Priority Score**	Genome with the highest user-defined priority score
	**(R)**	32	Genome indicated **RefSeq-representative** by the **NCBI**
	**(T)**	16	Genome from a type strain, indicated by the **NCBI**
	**(C)**	8	Genome with an assembly level estimated Complete
	**(S)**	4	Genome with an assembly level estimated Scaffold
	**(U)**	2	Genome with an assembly level estimated Unassembled
	**(d)**	-1	Genome whose quality is questionable
**2**	**Protein family distribution**		Genome with the largest distribution across protein families used as input, i.e. with the fewest missing values
**3**	**Centrality**		Genome whose Lin-clusters are the most frequent in the concerned MPS-cluster
**4**	**Pseudo-randomization**		Genome with the smallest hashing value

Additional file 11 – Centrality criterium

This Additional file will provide the proof that central genomes according to the Dice index can be chosen according to the frequency of their Lin-clusters, i.e. the size of the Lin-clusters to which their sequences belong.

Definition 1: In a set of genomes, the **central genome(s)** is/are the genome(s) whose average Dice index with all the other genomes is the greatest.

In other words, in a set of genomes, centrality is defined by the Dice index.

Theorem 1: In a set of genomes, the central genomes are the genomes whose Lin-clusters are the most frequent.

Introduction

Let's consider M genomes and N protein families. For each genome i and each protein family j, a sequence s_j^i belongs to a Lin-cluster x_j^i. Let's see an example with $M = 4$ genomes (g_A, g_B, g_C, g_D) and $N = 3$ protein families (f_1, f_2, f_3). It gives a matrix (x_j^i) of 4 rows and 3 columns.

$N = 3$ (columns); $M = 4$ (rows)

	f_1	f_2	f_3
g_A	1	1	1
g_B	1	1	1
g_C	1	1	2
g_D	2	1	3

$s_3^D : x_3^D = 3$

Lin-combination matrix

For example, the sequence s_3^D comes from the genome g_D, is attached to the protein family f_3 and was put in the Lin-cluster x_3^D which is labeled 3, i.e. $x_3^D = 3$.

Frequency of Lin-clusters

Now, for each Lin-cluster x_j^I, its frequency $freq(x_j^I)$ can be calculated. Noting the Dirac function δ, this frequency can be given by:

$$freq(x_j^I) = \frac{1}{M} \times \sum_{i=1}^{M} \delta(x_j^I, x_j^i)$$

Then an average frequency can be associated to each genome g_I by calculated the average of the frequency of its Lin-clusters. This average frequency can be given by:

$$freq(g_I) = \frac{1}{N} \times \sum_{j=1}^{N} freq(x_j^I) = \frac{1}{NM} \times \sum_{j=1}^{N} \sum_{i=1}^{M} \delta(x_j^I, x_j^i)$$

Let's see some applications from the previous example.

	f_1	f_2	f_3	$freq$
g_A	$3/4$	$4/4$	$2/4$	$9/12$
g_B	$3/4$	$4/4$	$2/4$	$9/12$
g_C	$3/4$	$4/4$	$1/4$	$8/12$
g_D	$1/4$	$4/4$	$1/4$	$6/12$

Average frequency of Lin-clusters

For example, the Lin-cluster $x_3^D = 3$ of the protein family f_3 appears once out of four genomes (g_D), so its frequency is equal to $freq(x_3^D) = 1/4$. Another example is the Lin-cluster $x_1^A = 1$ of the protein family f_1. It appears in three genomes out of four (g_A, g_B et g_C), so its frequency is $freq(x_1^A) = 3/4$. Last, the genome g_A has three Lin-clusters with the respective frequencies 3/4, 4/4 and 2/4, so the average frequency of the Lin-clusters of this genome g_A is:

$$freq(g_A) = \frac{1}{3} \times (\frac{3}{4} + \frac{4}{4} + \frac{2}{4}) = \frac{9}{12}$$

Dice index and centrality

Now, let's introduce the Dice index between the Lin-clusters of two genomes. In this context, the Dice index counts the proportion of common Lin-clusters between two genomes. The Dice index is given by the formula:

$$Dice(g_A, g_B) = \frac{1}{2\mathrm{N}} \times \sum_{j=1}^{N} 2\delta(x_j^A, x_j^B) = \frac{1}{\mathrm{N}} \times \sum_{j=1}^{N} \delta(x_j^A, x_j^B)$$

Let's see what happens for the previous example.

	g_A	g_B	g_C	g_D	$centrality$
g_A	$6/6$	$6/6$	$4/6$	$2/6$	$18/24$
g_B	$6/6$	$6/6$	$4/6$	$2/6$	$18/24$
g_C	$4/6$	$4/6$	$6/6$	$2/6$	$16/24$
g_D	$2/6$	$2/6$	$2/6$	$6/6$	$12/24$

Dice index matrix

For example, the two genomes g_A and g_B share identical Lin-clusters for the three protein families: $(1, 1, 1)$ so their Dice index is $Dice(g_A, g_B) = 6/6$. On the contrary, the two genomes g_C and g_D share only one Lin-cluster, that of the second protein family f_2, so their Dice index is: $Dice(g_A, g_B) = 2/6$.

Next, the centrality of a genome can be defined by its average Dice index to all the genomes (including itself). It can be given by the formula:

$$centrality(g_A) = \frac{1}{\mathrm{M}} \times \sum_{i=1}^{M} Dice(g_A, g_i) = \frac{1}{\mathrm{MN}} \times \sum_{i=1}^{M} \sum_{j=1}^{N} \delta(x_j^A, x_j^i)$$

The following lemma will link the centrality of a genome and the frequency of its Lin-clusters.

Lemma 1: The centrality of a genome is equals to the average frequency of its Lin-clusters, i.e.:

$$centrality(g_A) = freq(g_A)$$

Proof:

$$centrality(g_A) = \frac{1}{\mathrm{M}} \times \sum_{i=1}^{M} Dice(g_A, g_i) = \frac{1}{\mathrm{MN}} \times \sum_{i=1}^{M} \sum_{j=1}^{N} \delta\left(x_j^A, x_j^i\right)$$

The two sums can be switched because the sum is finite.

$$centrality(g_A) = \frac{1}{\mathrm{NM}} \times \sum_{j=1}^{N} \sum_{i=1}^{M} \delta\left(x_j^A, x_j^i\right) = \frac{1}{N} \times \sum_{j=1}^{N} freq\left(x_j^A\right) = freq(g_A)$$

□

Then the following theorem will be easily proved.

Theorem 1: In a set of genomes, the central genomes are the genomes whose Lin-clusters are the most frequent.

Prof: It is simply because the centrality of a genome is equal to the average frequency of its Lin-clusters. So the genome whose Lin-clusters have the highest average frequency will also be the genome with the higher centrality.

□

Corollary 1: The most central genomes can be chosen according to the frequency of the Lin-clusters. The genomes whose Lin-clusters have the highest average frequency will be the most central, according to the Dice index.

In the previous example, the most central genomes are g_A and g_B because the average frequency of their Lin-clusters and their centrality will be the higher, i.e. $\frac{9}{12} = \frac{18}{24}$.

Additional file 12 – Preparation of the bacterial dataset

The initial dataset corresponded to 55 protein families from 200,565 bacterial genomes from the database RiboDB [10]. These 55 protein families were the following: bl12, bl17, bl19, bl20, bl21, bl25, bl27, bl28, bl31, bl32, bl33, bl34, bl35, bl36, bl9, bs16, bs18, bs20, bs21, bs6, bTHX, cs23, ul1, ul10, ul11, ul13, ul14, ul15, ul16, ul18, ul2, ul22, ul23, ul24, ul29, ul3, ul30, ul4, ul5, ul6, us10, us11, us12, us13, us14, us15, us17, us19, us2, us3, us4, us5, us7, us8, us9. Multicopy sequences in a genome were omitted. The dataset included 9,693,984 single-copy sequences.

7 families bl25, bl31, bl33, bl36, bTHX, cs23 and us14 were omitted because present in less than 50% of the genomes. 22,362 genomes were omitted because containing less than 80% of their sequences. 86,480 sequences were omitted because of abnormal length (smaller than 75% of the median length or greater than 125% of the median length).

8,315,939 single-copy sequences were analyzed, corresponding to 178,203 genomes and 48 protein families. These 48 protein families were the following: bl12, bl17, bl19, bl20, bl21, bl27, bl28, bl32, bl34, bl35, bl9, bs16, bs18, bs20, bs21, bs6, ul1, ul10, ul11, ul13, ul14, ul15, ul16, ul18, ul2, ul22, ul23, ul24, ul29, ul3, ul30, ul4, ul5, ul6, us10, us11, us12, us13, us15, us17, us19, us2, us3, us4, us5, us7, us8, us9.

Additional file 13 – Generation of the artificial bacterial dataset

The bacterial dataset has been used to simulate artificial genomes. In the example below, 4 bacterial genomes (**g_A, g_B, g_C, g_D**) have been used to generate 4 artificial genomes (**g_A', g_B', g_C', g_D'**). Each bacterial genome is used as a receiver and receives a gene from another genome, used as donor. In the example below, the genome **g_A** is used as receiver genome and receives the gene **s_C^3** through an horizontal gene transfer from the genome **g_C** used as donor. This process is replicates three times meaning that each genome from the bacterial dataset will be used to generate three artificial genomes (**g_A', g_A'', g_A'''**). As a result, the 178,203 x 3 = 534,609 artificial genomes have been generated from the 178,203 natural genomes.

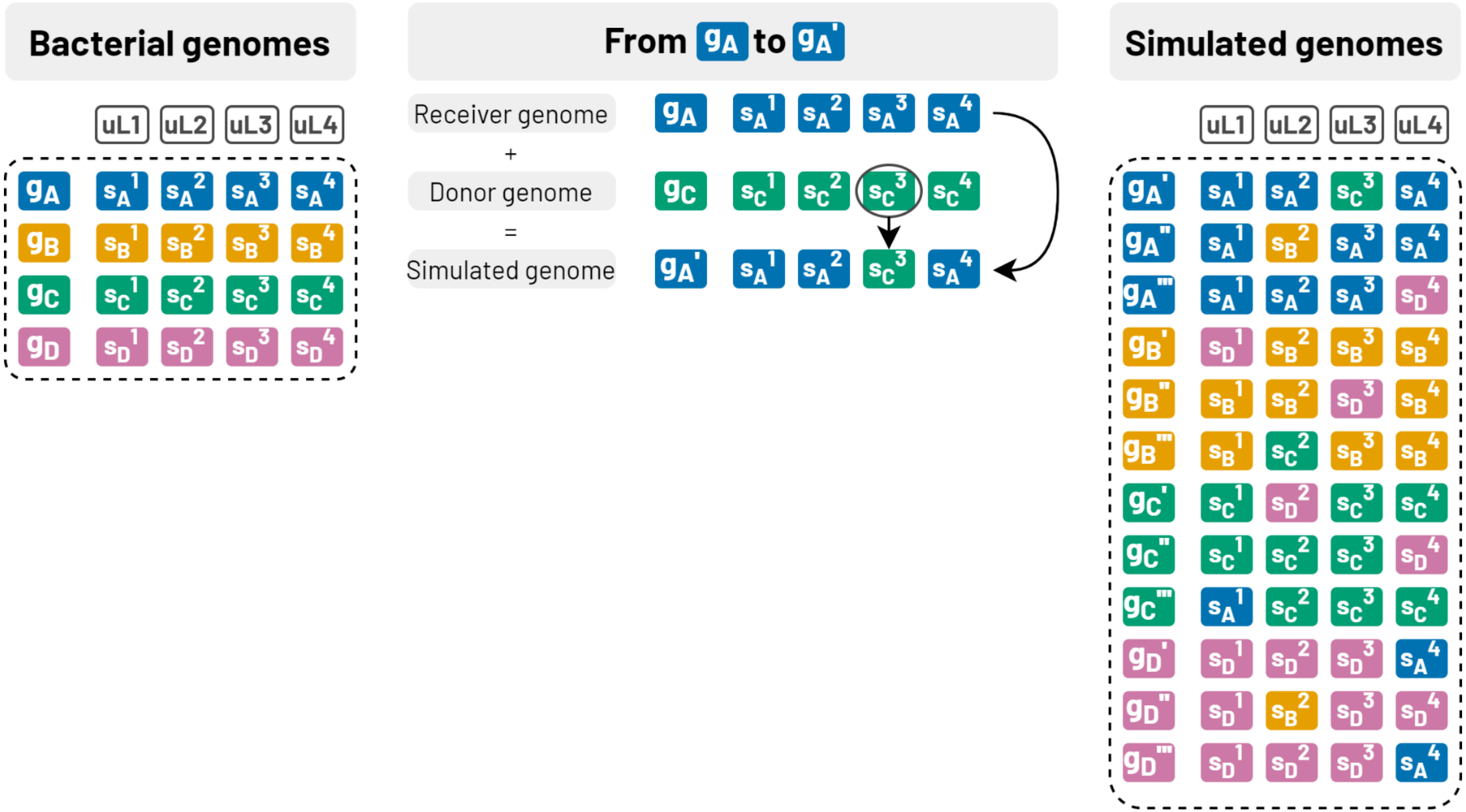

Additional file 14 - Choice of the parameters of MPS-Sampling (without pre-connection)

Step 1: Protein clustering (eValue = 10^{-5}, coverageMode = 0, minCov = 0.8, and minSeqID = 0.6)

For the construction of the Lin-clusters (Step 1), Linclust parameters were set as follow: eValue = 10^{-5}, coverageMode = 0, minCov = 0.8, and minSeqID = 0.6. This corresponded to the code for Linclust: "-e 0.00001 -cov-mode-0 -c 0.8 –min-seq-id 0.6". The coverage mode 0 corresponded to a bidirectional coverage between query and target. The step involving Linclust lasted 1 minute without parallelization (Additional file 24) and could be automatically parallelized with the Snakemake pipeline if needed. The Linclust parameters were tested. Only the minimum sequence identity seems to significantly impact the number of Lin-clusters (Additional files 15-18). This value of minSeqID = 0.6 brought a median of 773 Lin-clusters along the 48 proteins (Additional file 15), which represent roughly the number of taxonomic families of 661. Based on these parameters, within each Lin-cluster, protein sequences were expected to share at least half of their amino acid positions with the centroid sequences (minSeqID x minCov = 0.60 x 0.80 = 0.48). To resume, Linclust parameters were set as follow: eValue = 10^{-5}, coverageMode = 0, minCov = 0.8, and minSeqID = 0.6.

Step 2: Construction of elementary groups of genomes (EGG) and of the Lin-combination matrix (no parameter)

For the construction of the Lin-combinations and the elementary groups of genomes (EGG) (Step 2), there is no parameter to adjust the construction of the EGG.

Step 3: No pre-connection was applied

For pre-connection (Step 3), this step was skipped for the main run of MPS-Sampling so no parameter needed to be chosen.

Step 4: Construction of MPS-clusters

For the construction of the MPS-clusters (Step 4), the minimum similarity Δ was set to eleven different values $\Delta \in \{1, 0.9, 0.8, 0.7, 0.6, 0.5, 0.4, 0.3, 0.2, 0.1, 0.05\}$, leading to eleven different runs and thus to eleven samplings of different sizes.

Additional file 15 – Median number of Lin-clusters according to minimum sequence identity

Univariate study of the median number of Lin-clusters according to the minimal sequence identity (**minSeqID**) with the fixed parameters: **covMode = 0**, **eValue = 10e-5** and **minCov = 0.8**.

Three lines represent the median number (blue) as well as the first and third quartiles (grey) of Lin-clusters. As an indication, three green lines represent the number of species, genera and families present in the dataset.

The minimum sequence identity had clearly an important impact on the cluster finesse and constitutes the major parameter to fine-tune for Linclust. By fixing it to **minSeqID = 0.6**, the median number of Lin-clusters (773) approached the number of taxonomic families (661).

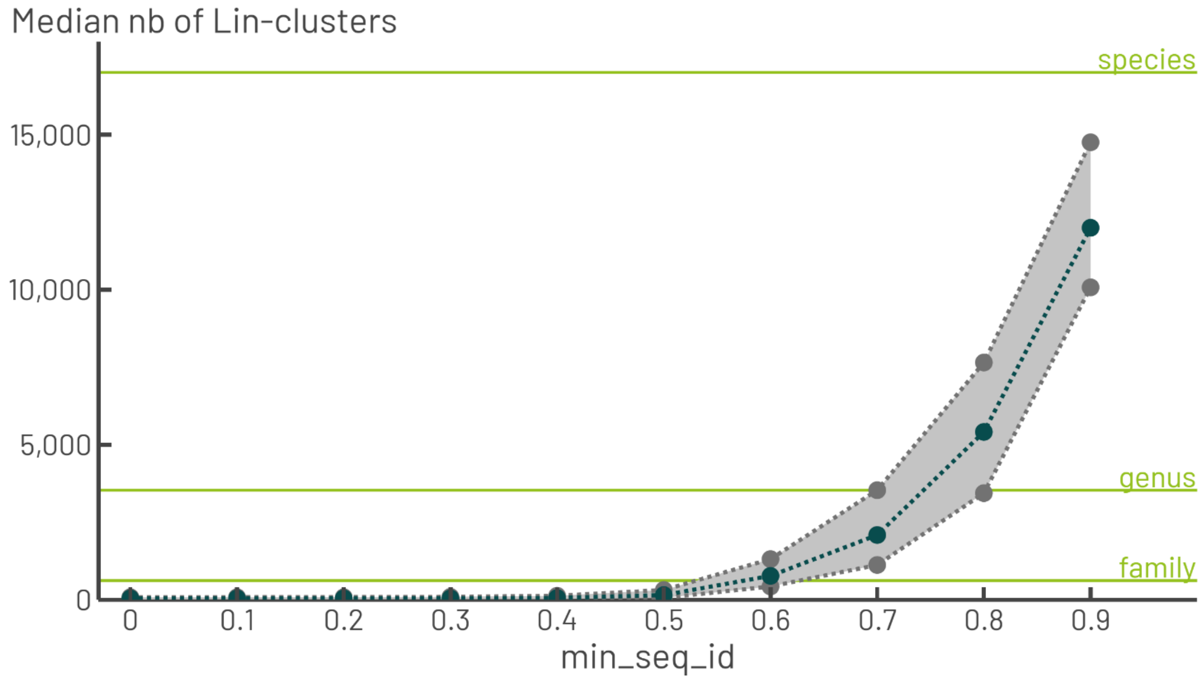

Additional file 16 – Median number of Lin-clusters according to coverage mode

Univariate study of the median number of Lin-clusters according to the coverage mode (**covMode**) with the fixed parameters: **eValue = 10e-5**, **MinCov = 0.8** and **minSeqID = 0.6**.

Three lines represent the median number (blue) as well as the first and third quartiles (grey) of Lin-clusters. As an indication, three green lines represent the number of species, genera and families present in the dataset.

The line is almost flat, so the coverage mode did not have a large impact on the sequence cluster finesse.

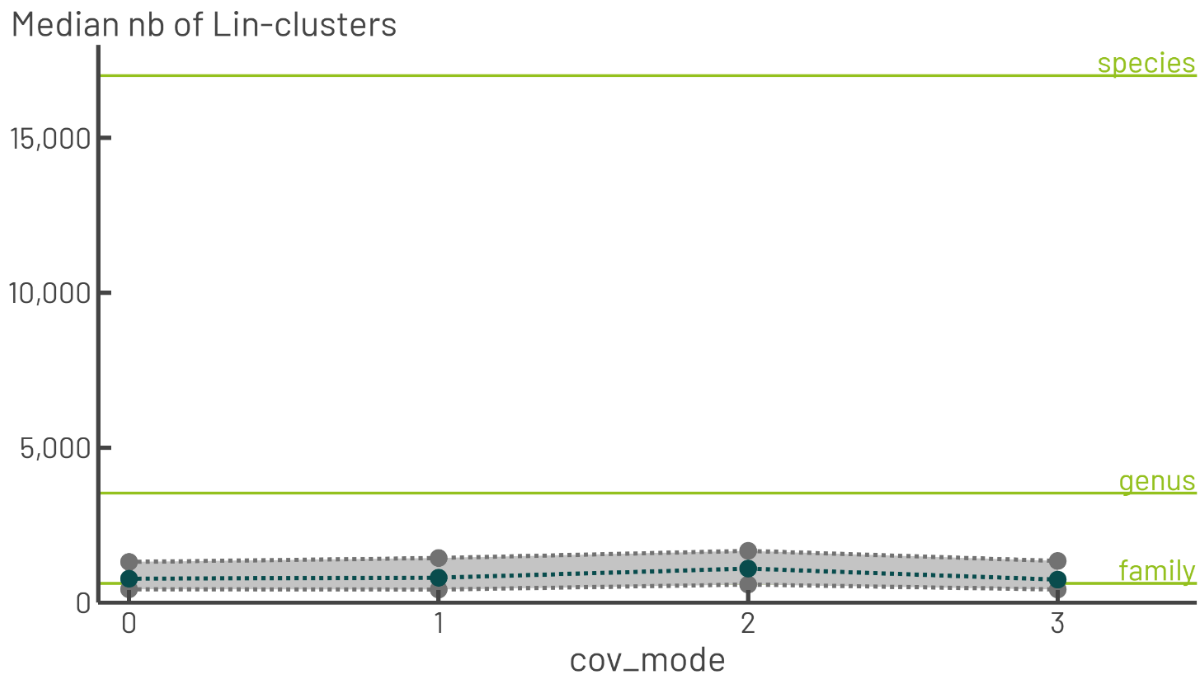

Additional file 17 – Median number of Lin-clusters according to eValue

Univariate study of the median number of Lin-clusters according to the **eValue** with the fixed parameters: **covMode = 0**, **minCov = 0.8** and **minSeqID = 0.6**.

Three lines represent the median number (blue) as well as the first and third quartiles (grey) of Lin-clusters. As an indication, three green lines represent the number of species, genera and families present in the dataset.

The line is completely flat, so the eValue had almost no impact on the sequence cluster finesse.

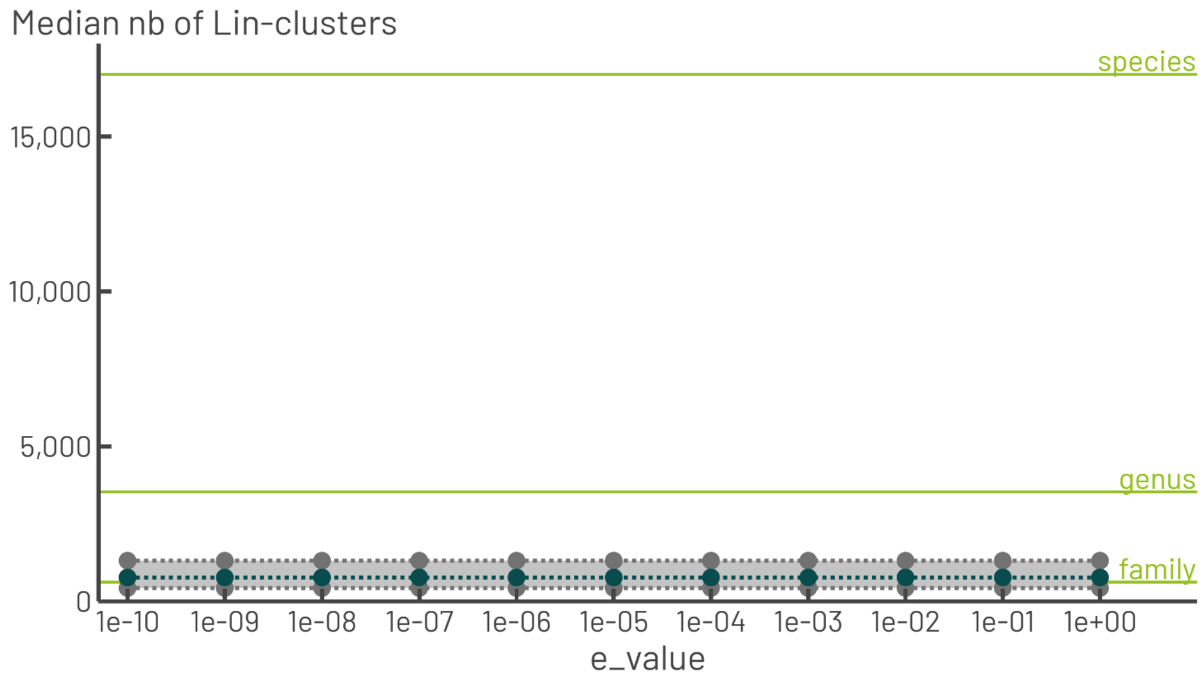

Additional file 18 – Median number of Lin-clusters according to minimum coverage

Univariate study of the median number of Lin-clusters according to the minimal coverage (**minCov**) with the fixed parameters: **covMode = 0**, **eValue = 10e-5** and **minSeqID = 0.6**.

Three lines represent the median number (blue) as well as the first and third quartiles (grey) of Lin-clusters. As an indication, three green lines represent the number of species, genera and families present in the dataset.

The minimum coverage had almost no impact from 0.3 to 0.7. From 0.8 to 0.9, it had a little impact.

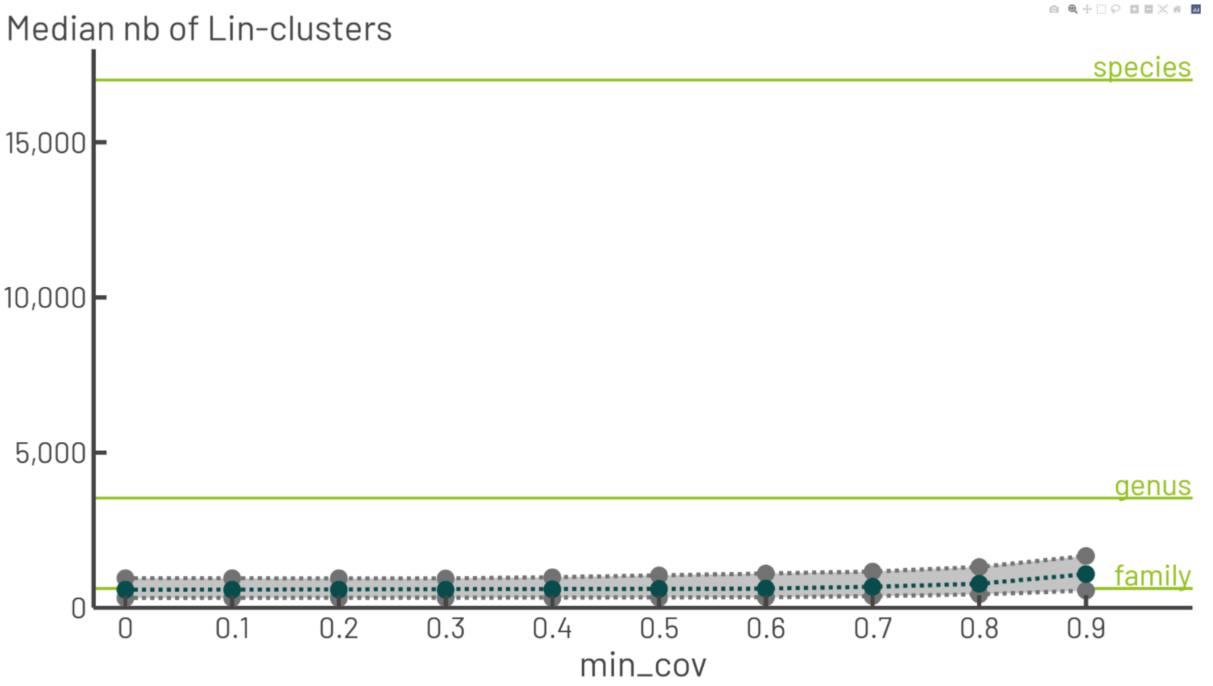

Additional file 19 – Choice of the parameters of MPS-Sampling (for pre-connection)

When using the pre-connection (Step 3), no other parameter was changed, as described in the Additional file 14. The pre-Connection was very fast (2 minutes) (data not shown), allowing to easily test different values for the unique parameter MinNbLinclusters .

The parameter was set to **MinNbLinclusters = 25**. It leaded to trade-off between the number of pre-connected components (488) (Additional file 20) and the size of the largest pre-connected component, which encompassed 25,351 Lin-combinations (Additional file 21).

Additional file 20 – Size of the largest pre-connected component depending on MinNbLinclusters

The decreasing of the curve is coherent because the greater the MinNbLinclusters, the more the overall genomic dataset was divided and the smaller the largest pre-connected component.

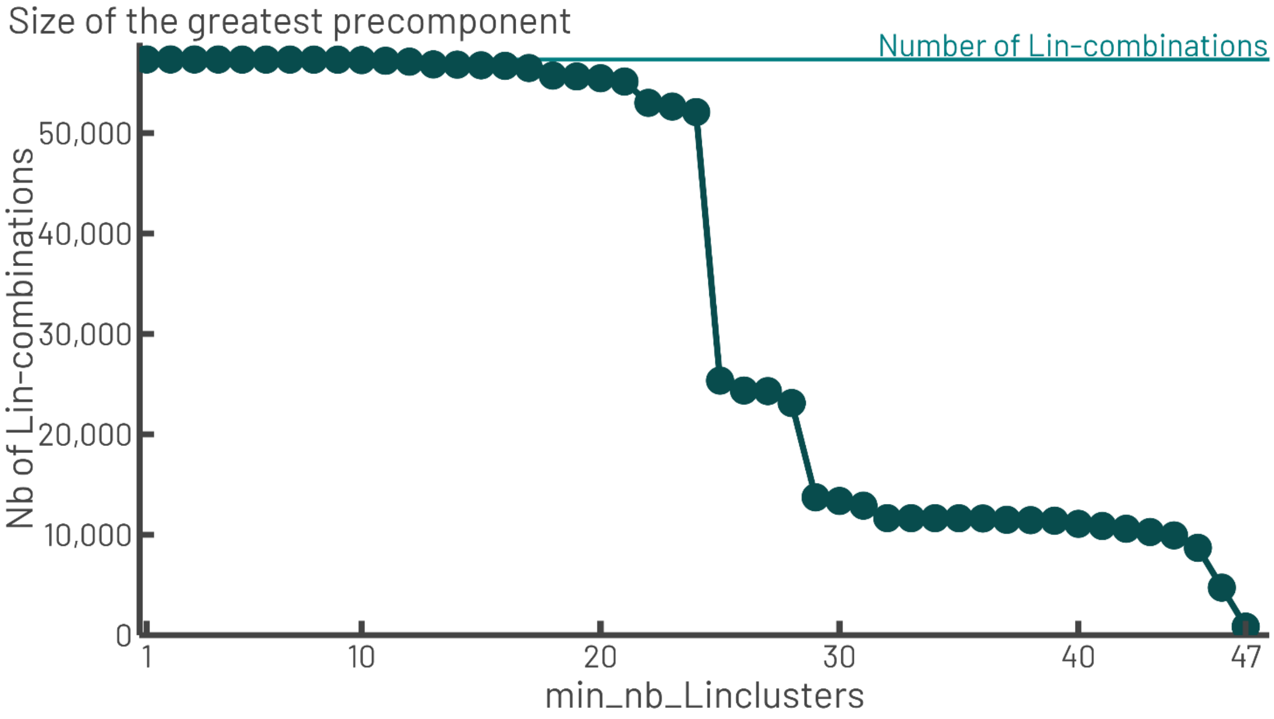

Additional file 21 – Number of pre-connected components depending on MinNbLinclusters

The decreasing of the curve is coherent because the greater the MinNbLinclusters, the more the overall genomic dataset was divided, and the more pre-connected components there were.

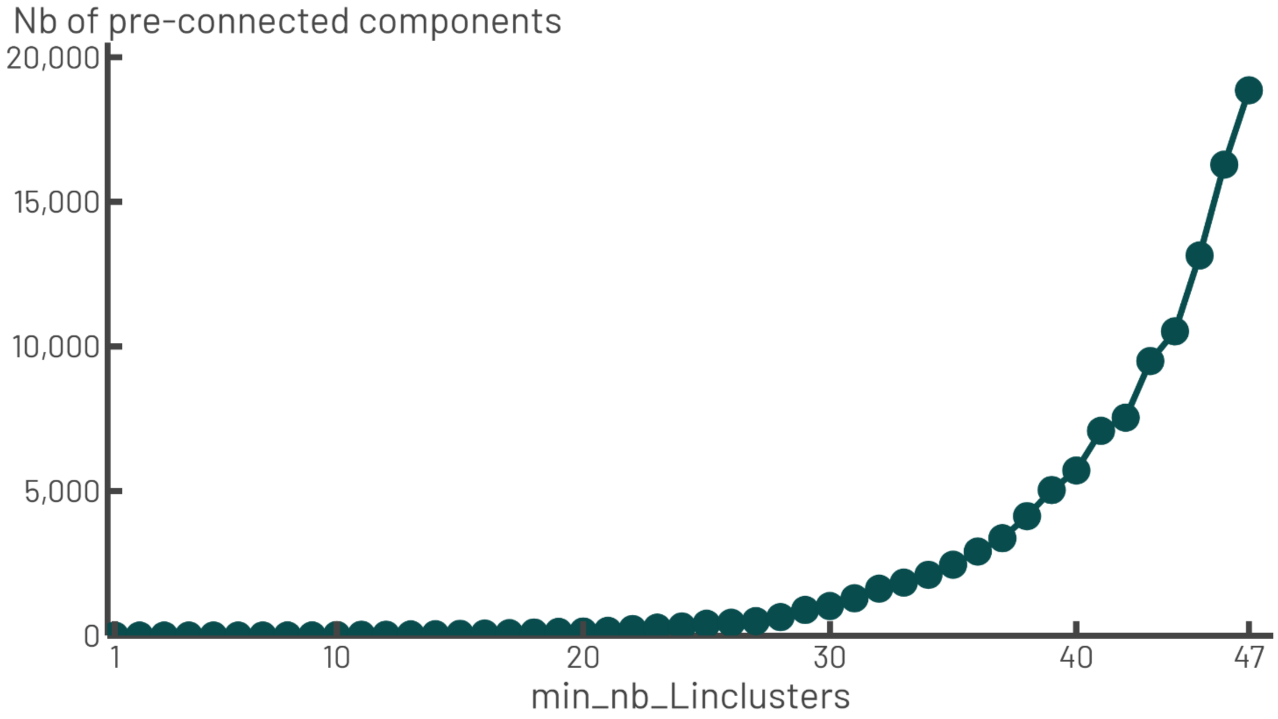

Additional file 22 - Phylogenetic reconstruction

For phylogenetic inference, for each of the 48 studied r-prot families, protein sequences were aligned with kalign v3.2.2 (March 22) [11] with default parameters. The resulting alignments were trimmed by removing positions containing more than 30% of gaps and sequences with more than 30% of gaps. Trimmed alignments were combined to build a large supermatrix. A second trimming round has been performed on the supermatrix to remove the columns of positions containing more than 20% of gaps and sequences containing more than 20% of gaps. These filters are used to limit the amount of missing data in the supermatrices that may bias phylogenetic reconstructions [12]. Phylogenies were reconstructed using FastTree v2.1.11 (Feb 20) [13] with default parameters. The phylogeny inference of the 178,203 genomes of the bacterial dataset lasted 51.50 hours with a parallelization using 3 cores on a server with 32 cores and 64 threads (AMD EPYC 7542 32 Core Processor @3,40Ghz) and 1 To of DDR4 under Debian Trixie.

Additional file 23 – TaxSampler, a homemade-software for sampling genomes based on taxonomy

TaxSampler is an unpublished homemade-software that samples genomes according to their taxonomic affiliation. The taxonomic affiliation that has been used comes from the NCBI. It can be applied to any taxonomic level: phylum, class, order, family, genus, or species. TaxSampler uses as input a list of genomes with taxonomy and provides as output a list of representative genomes for the chosen taxonomic level, called Tax-representatives.

- TaxSampler uses as input a list of genomes with taxonomy.
- TaxSampler discards genomes whose taxonomic affiliation of the chosen taxonomic level is unknown.
- TaxSampler groups genomes according to the taxonomic affiliation of the chosen taxonomic level.
- TaxSampler choses a representative genome, called Tax-representative, within each group. This Tax-representative is chosen according to the first and fourth priority rules of MPS-Sampling (Additional file 10). First, the genomes with the highest priority score are chosen. Second, the genome with the smallest hashing value is chosen.

Additional file 24 – Computational time MPS-Sampling concerning the 178,203 genomes of the bacterial dataset

MPS-sampling was run without pre-connection through a single-threaded process on a shared server with 32 cores and 64 threads (AMD EPYC 7542 32 Core Processor @3,40Ghz) and 1 To of DDR4 under Debian Trixie. The computational time is measured with elapsed time and has been rounded to the minute.

The preparation of the data lasted 12 minutes: 3 minutes for the retrieval and the download of the data from the RiboDB website (https://umr5558-bibiserv.univ-lyon1.fr/riboDB/ribodb.cgi), 5 minutes for the filter of the genomes and of the protein sequences (as described in the Additional file 12) and 4 minutes for the formatting of the FASTA files (essentially renaming the sequence labels with the accession number of the corresponding genome).

The computational time required to generate one MPS-sample was 4 + 1 + 51 + 1 + 2 = 59 min. Because the steps 1 to 4-1 are common to several MPS-samples, the computational time for eleven MPS-samples was 4 + 1 + 51 + (1 + 2) * 11 = 89 min = 1h29.

The most time-consuming step was the computing of similarity matrix (step 4-1) due to quadratic complexity. This limitation can be partially relaxed using pre-connection, saving a substantial amount of time (see Results and Additional file 25).

Algorithm	Step	Task	Computational time
MPS-Sampling	Step 1	Protein clustering	4 min
MPS-Sampling	Step 2	Construction of elementary groups of genomes (EGG) and of the Lin-combination matrix	1 min
MPS-Sampling	Step 3	None (skipped step)	
MPS-Sampling	Step 4-1	Computation of the similarity matrix	51 min
MPS-Sampling	Step 4-2	Construction of MPS-clusters	1 min
MPS-Sampling	Step 5	Selection of MPS-representatives	2 min

Additional file 25 – Computational time

Here are four examples about MPS-Sampling and computational time. In each case, MPS-Sampling was always launched to generate one sample with Δ = 0.4 through a single-threaded process.

A: Impact of the machine

Running time of MPS-Sampling on the bacterial dataset (178,203 genomes) was measured on two different servers:

- **PkDBServ:** A dedicated server with 20 cores and 40 threads (2 Intel Xeon E5 2660v2 CPUs @2.20Ghz) and 128 GB of DDR3 RAM running Debian 10 (Buster).
- **AmalphyLab:** A shared server with 32 cores and 64 threads (AMD EPYC 7542 32 Core Processor @3.40Ghz) and 1 TB of DDR4 RAM running Debian Trixie.

The difference is significant. With the 178,203 genomes, the running time was 30% higher with PkDBServ than with AmalphyLab (respectively 4,520 secs and 3,472 secs).

B: Impact of pre-connection

The bacterial dataset (178,203 genomes and48 rprot families) was analyzed with and without pre-connection. With pre-connection, the analysis was much faster. With the 178,203 genomes, the run was 70% faster with pre-connection than without (respectively 1,030 secs and 3,472 secs).

C: Comparison MPS-Sampling and Treemmer

Treemmer was much slower than MPS-Sampling. With the 178,203 genomes, Treemmer lasted 360h, that was 360 times slower than MPS-Sampling.

D: Impact of the size of the dataset

Running elapsed time was measured up to 534,609 ABD genomes (48 r-prot families). It was compared with the running time of the 178,203 bacterial genomes (48 r-prot families). In the top left-hand corner, a zoom between 0-150k genomes shows that the running time was similar between the true genomes and the ABD genomes. The relation between the number of ABD genomes and the running time was estimated. According to a regression model, the relation was $T = 1.30 * 10^{7} * N$ where T is the computational elapsed time in seconds and N the number of analyzed genomes (p-value <2e-16).

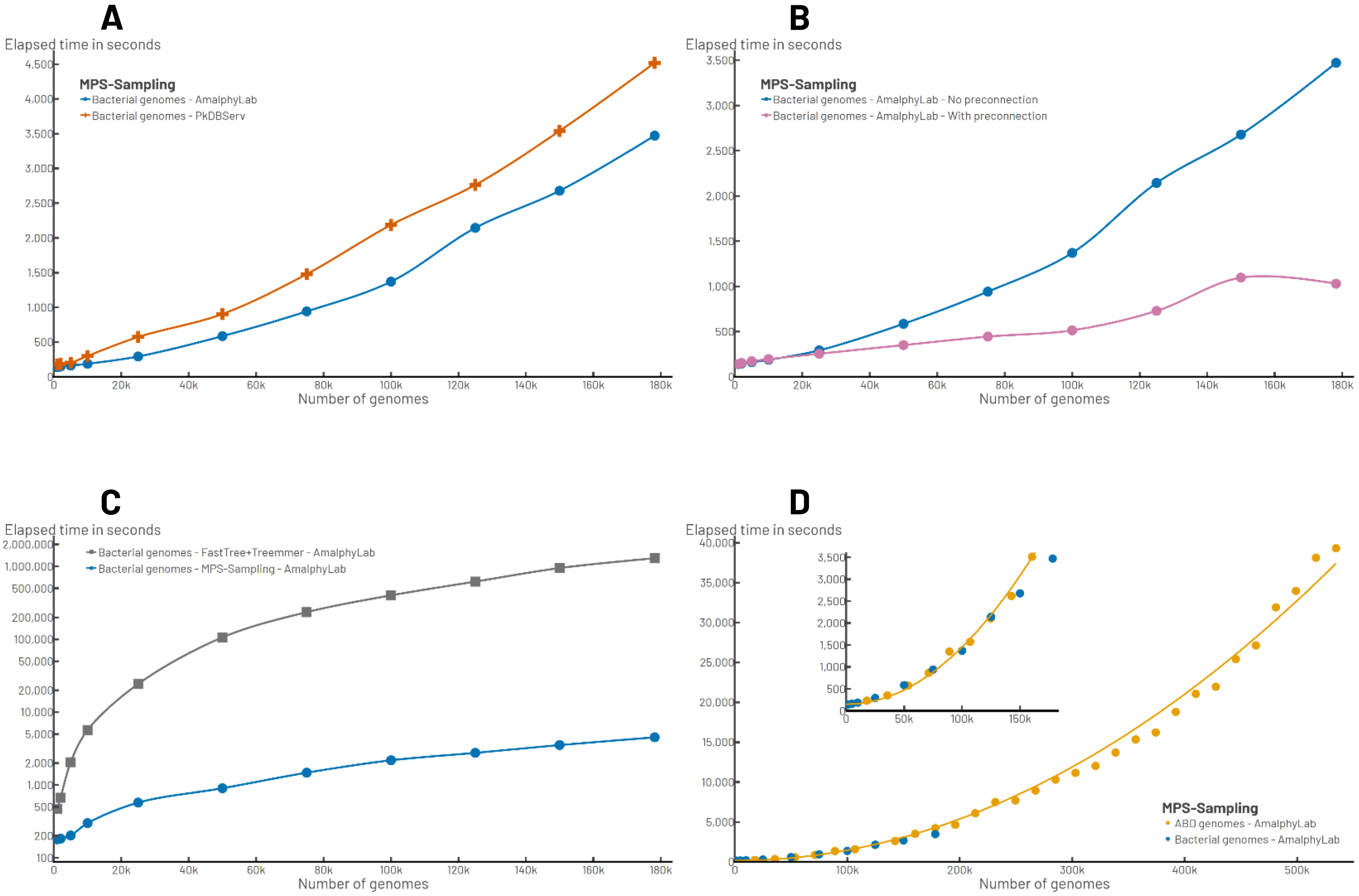
A
Elapsed time in seconds
MPS-Sampling
Bacterial genomes - AmalphyLab
Bacterial genomes - PkDBServ
Number of genomes
B
Elapsed time in seconds
MPS-Sampling
Bacterial genomes - AmalphyLab - No preconnection
Bacterial genomes - AmalphyLab - With preconnection
Number of genomes
C
Elapsed time in seconds
Bacterial genomes - FastTree+Treemmer - AmalphyLab
Bacterial genomes - MPS-Sampling - AmalphyLab
Number of genomes
D
Elapsed time in seconds
MPS-Sampling
ABD genomes - AmalphyLab
Bacterial genomes - AmalphyLab
Number of genomes

Additional file 26 – Intermediate results of MPS-Sampling concerning the bacterial dataset

Step 1: Protein clustering (eValue = 10^{-5}, coverageMode = 0, minCov = 0.8, and minSeqID = 0.6)

Starting from 48 r-prot families from 178,203 bacterial genomes, 59 to 2,789 Lin-clusters per r-prot family were built (median = 773). This reflects differences in sequence variation (e.g. selective pressure) among r-prot families: the higher the identity among sequences within a protein family, the smaller the number of Lin-clusters. Below is presented the number of Lin-clusters per protein family.

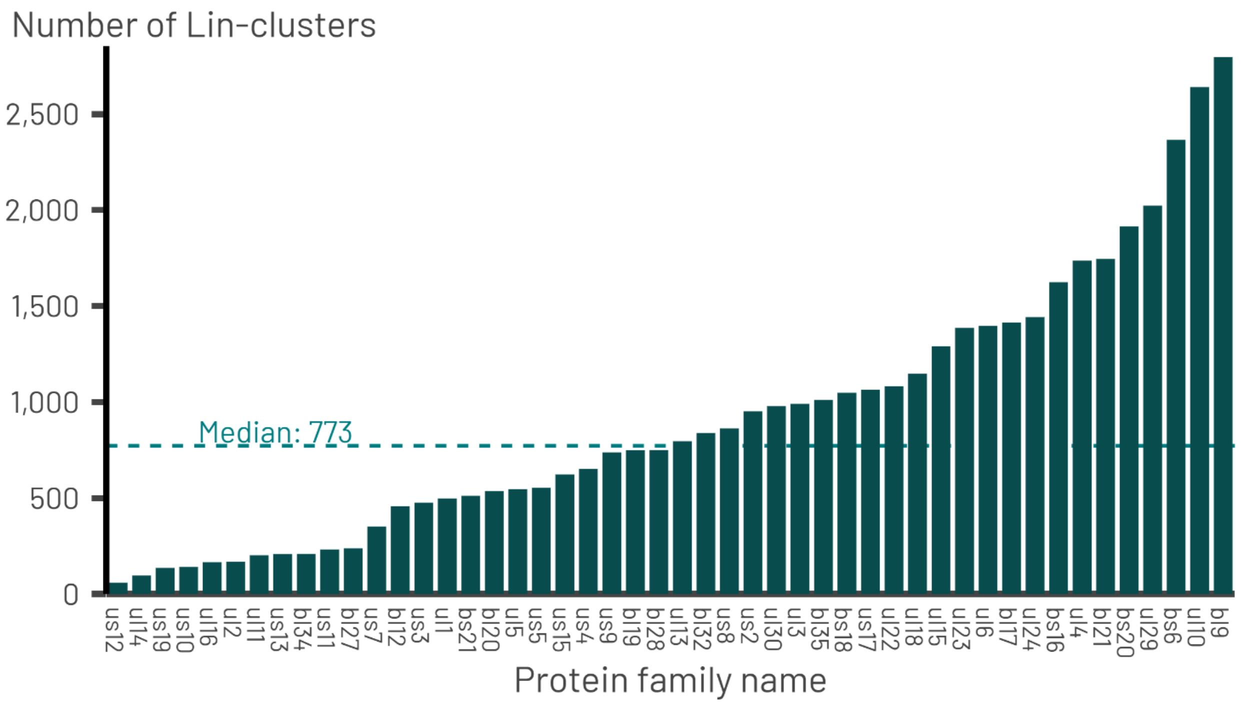

The number of Lin-clusters was not correlated with the length of the protein sequences. Below is presented the median length of protein sequences according to the number of Lin-clusters.

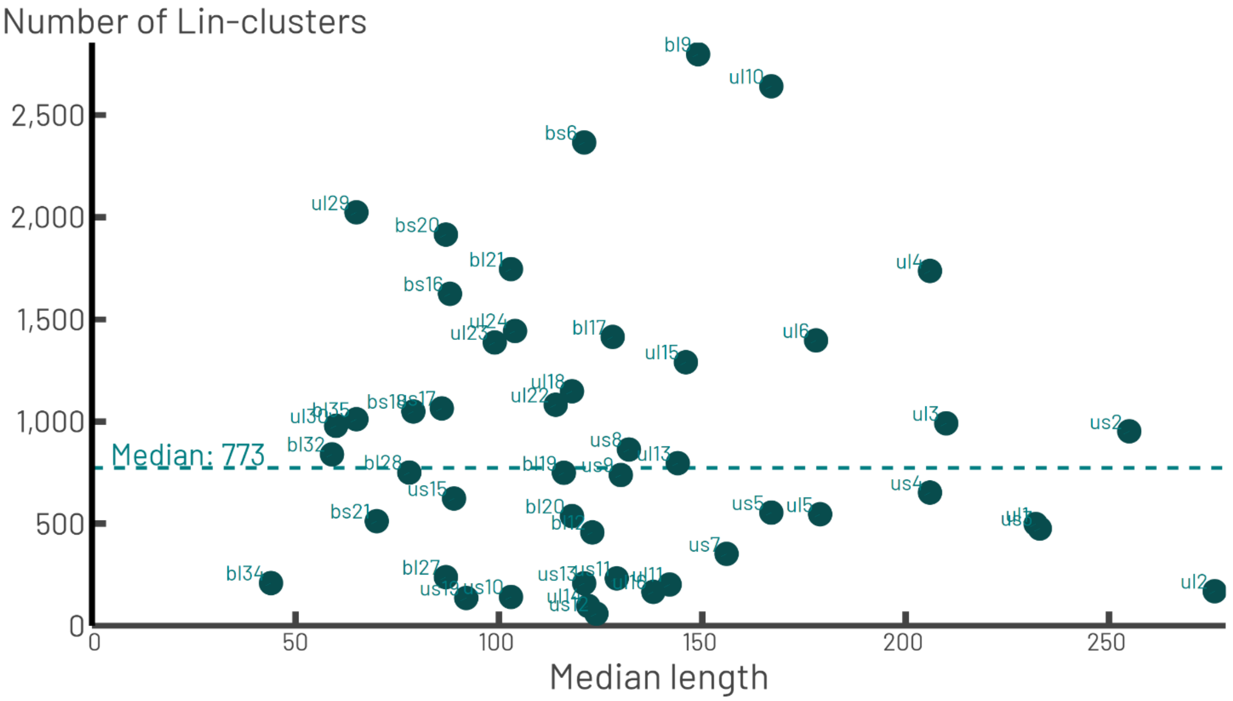

Step 2: Construction of elementary groups of genomes (EGG) and of the Lin-combination matrix (no parameter)

The Lin-clusters among the 178,203 bacterial genomes constituted 57,332 Lin-combinations among which 48,296 (84,8%) were singleton (i.e. contain a single genome). In contrast, the three largest encompassed 4.21% of initial dataset (i.e. 2,891, 2,575, and 2,037 genomes, respectively). Below is presented the number of Lin-combinations according to the number of genomes they contained.

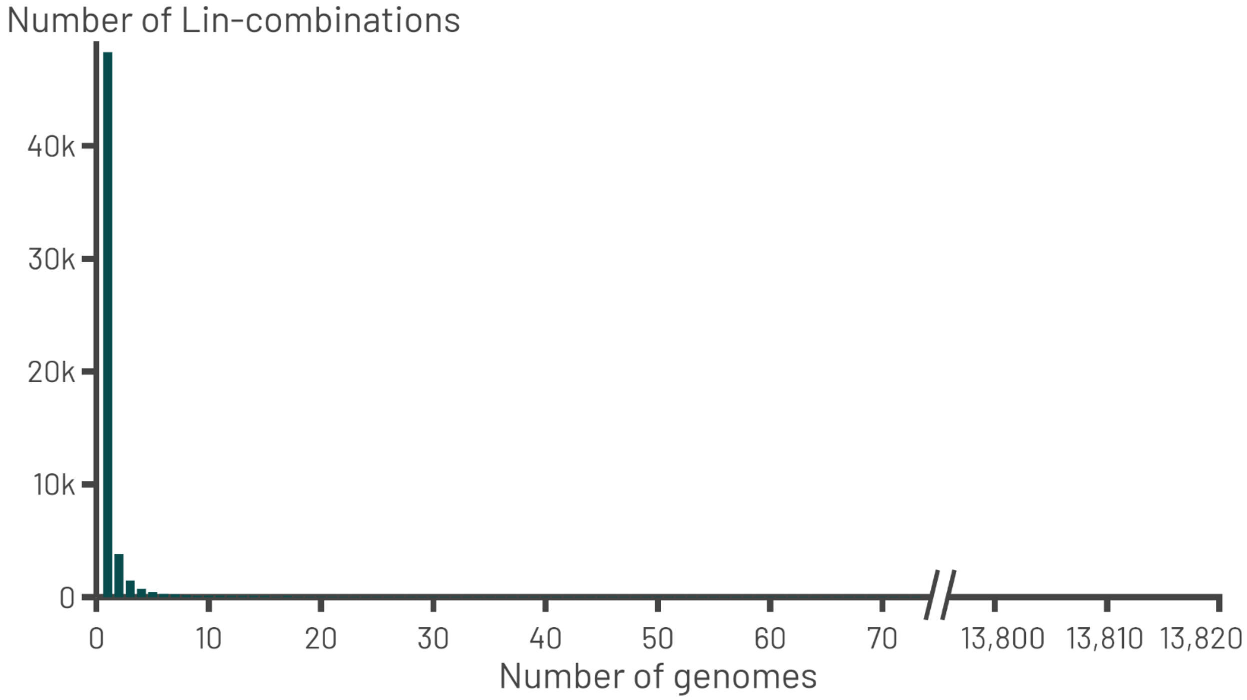

Step 3: No pre-connection was applied

Step 4: Construction of MPS-clusters (Δ = 0.7)

When Δ = 0.7, 12,787 MPS-clusters were built, among which 5,329 contained a single genome and 2,404 contained two genomes, while the two largest gather 13,820 and 3,385 genomes, respectively.

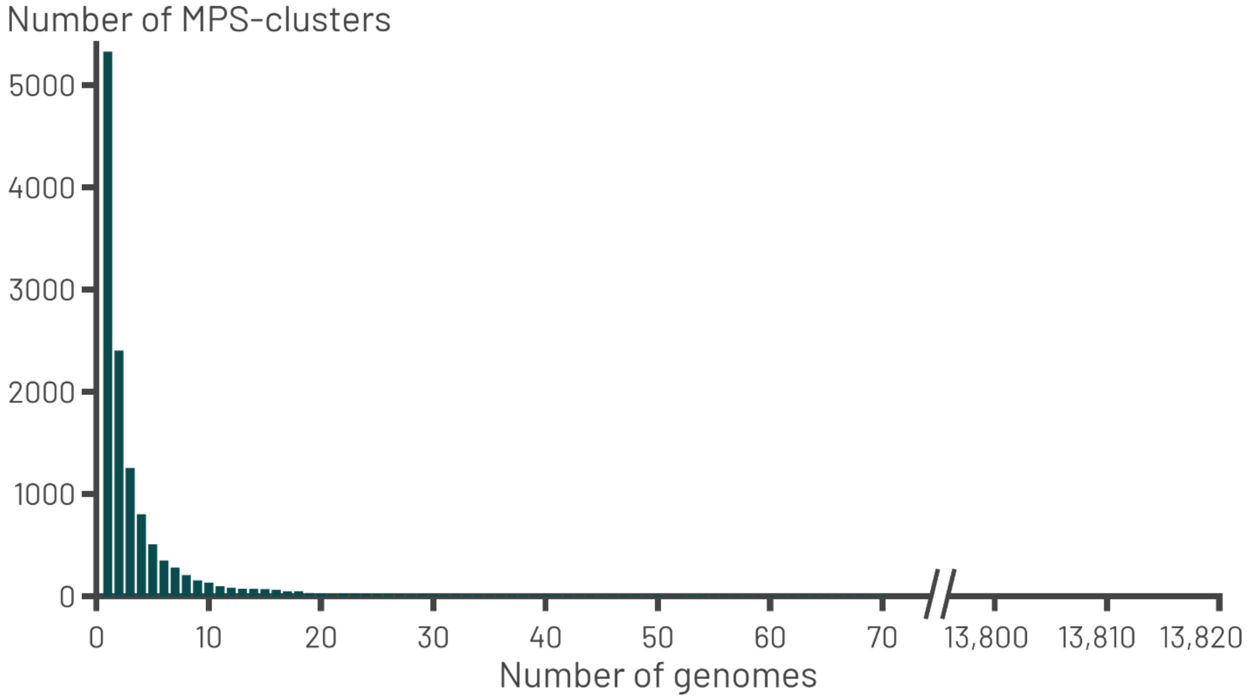

Additional file 27 – Number of MPS-clusters where each selection rule was applied

With Δ=0.7, 12,787 MPS-clusters were generated; the frequency of the four priority rules for the choice of the MPS-representatives (Additional file 10) will be detailed. Among the 12,787 generated MPS-clusters, 12,787 - 7,446 = 5,341 MPS-clusters contain only one genome, so no selection rule needed to be applied. It meant that 7,446 MPS-clusters had more than one genome. The first rule (priority score) was applied to all these 7,446 MPS-clusters. After that, 4,833 MPS-clusters still had more than one genome, so the second rule (protein distribution) was applied to these 4,833 MPS-clusters. Then, the third rule (centrality) was applied to 2,603 MPS-clusters. Finally, the last rule (pseudo-randomness) was applied to 817 MPS-clusters.

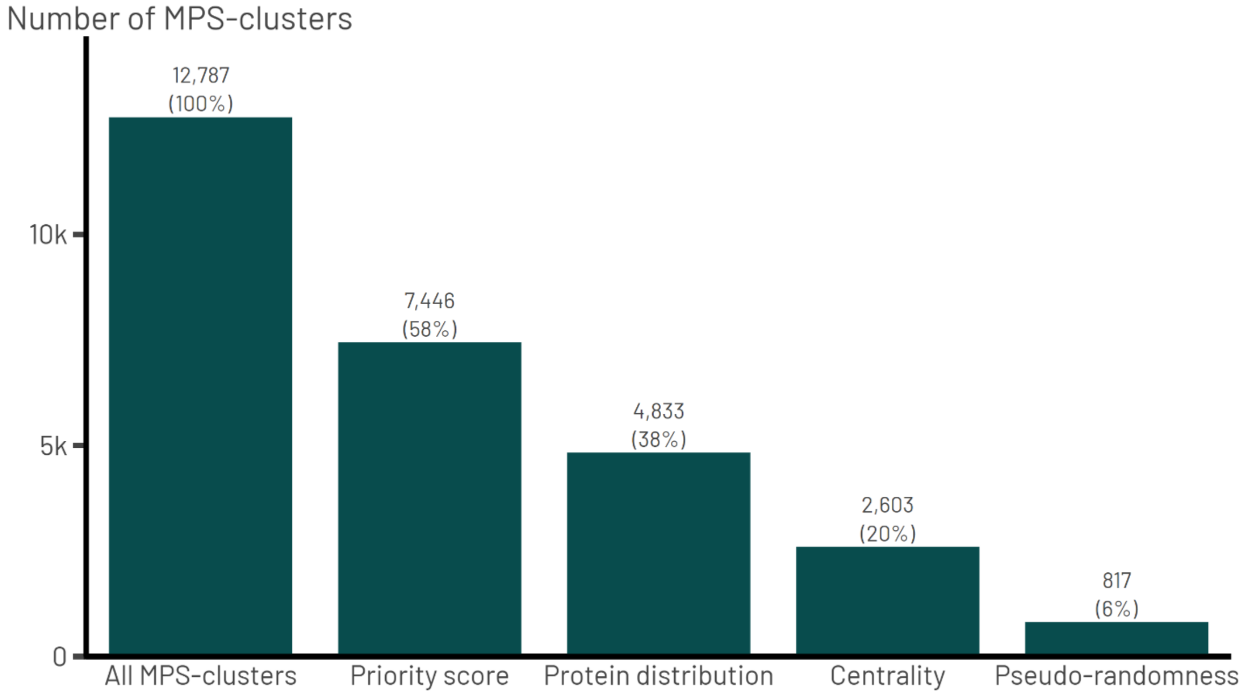

Additional file 28 – Taxonomic statistics about each investigated subset

MPS-Sampling was launched on the bacterial dataset, encompassing 178,203 bacterial genomes. In addition to the complete dataset, some subsets were investigated. The constitution of the bacterial reference, a subset encompassing 35,103 genomes, is described in the Additional file 30. Three taxonomic families have also been investigated, respectively *Lactobacillaceae Bacillaceae* and *Enterobacteriaceae*.

For example, the family *Lactobacillaceae* encompassed 6,410 genomes, including 4,992 (98%) with a complete nomenclature. It involves 361 species and 32 genera, with 11.28 species per genus in average. Concerning the taxonomic density, there were 13.83 and 159.72 genomes per species and genus in average, respectively.

Subset		Genome Number			Taxonomic Diversity		Taxonomic Density		
**Level**	**Name**	**Total**	**Having a complete nomenclature**		**Number of species** *(without sp. and subsp.)*	**Number of genera**	**Species per genus**	**Genomes per species** *(from complete nomenclature)*	**Genomes per genus** *(from complete nomenclature)*
domain	bacterial dataset	178,203	135,315	76%	16,814	3,896	4.32	8.12	40.95
domain	bacterial reference	35,103	16,836	48%	16,228	3,787	4.29	1.07	5.13
family	*Lactobacillaceae*	6,410	6,268	98%	379	33	11.48	16.56	194.18
family	*Bacillaceae*	7,113	6,160	87%	644	108	5.96	9.57	65.70
family	*Enterobacteriaceae*	17,096	15,692	92%	178	58	3.07	88.16	294.21

Additional file 29 – Phylogenic statistics about each phylogenetic inference

For each investigated group (level + group name), a set of genomes was used for phylogenetic inference (genomes number). After recruitment, alignment and trimming, a supermatrix was built with a given number of rows (sequences number) and columns (positions number); moreover, it had a given number of missing values (gaps number) and a given proportion of missing values among all cells (gaps ratio). From the computed phylogenetic tree, the sum of all branch lengths was divided by the number of tips; this quotient gave an indication about the phylogenetic diversity of the genomic set (total length / number of tips).

For example, the family *Lactobacillaceae* encompassed 6,410 genomes, i.e. 6,410 sequences (one sequence per genome) and 6,105 positions. So, the supermatrix had 6,410 rows and 6,105 columns. There were 1,020,435 gaps in the supermatrix, i.e. 2.61% of it. After the phylogenetic inference, the sum of all branch lengths divided by the number of tips was equal to 0.0026. This provides an indicator about the phylogenetic inference.

Subset		Supermatrix					Tree
**Level**	**Group Name**	**Genomes Number**	**Sequences Number**	**Positions Number**	**Gaps Number**	**Gaps Ratio**	**Total length / Number of tips**
domain	bacterial dataset	178,203	178,203	5,874	30,880,709	2.95%	0.0159
domain	bacterial reference	35,103	35,103	5,819	10,386,208	5.08%	0.0716
family	*Lactobacillaceae*	6,410	6,410	6,105	1,020,435	2.61%	0.0026
family	*Bacillaceae*	7,113	7,113	6,138	434,529	1.00%	0.0046
family	*Enterobacteriaceae*	17,096	17,096	6,188	514,233	0.49%	0.0008

Additional file 30 – Construction of a reference bacterial phylogeny

One way of assessing the quality of genome samples is to look at the phylogenetic position of the selected genomes in a tree containing all the genomes. The main difficulty is that it is not possible to visualize trees with a very large number of leaves. Thus, for visualization, the bacterial dataset of 178,203 genomes was reduced to a reference bacterial phylogeny of 35,159 genomes. More precisely, all the 16,135 RefSeq-representative genomes were kept, as they were considered as a standard against which other data should be compared [1]. For instance, when Δ= 0.7, these genomes were MPS-representatives and/or belonged to 4,909 MPS clusters, that together represent 159,179 genomes (78%). It was assumed that these 16,135 RefSeq-representative genomes were a reliable reference to represent these 159,179 genomes. The remaining 19,024 genomes (11%) were distributed across the 7,878 MPS clusters that did not contain any RefSeq-representative genomes. Because these genomes could not be linked to any reference external to our analysis, all of them were kept for the phylogenetic analysis. Thus, in total, 16,135 + 19,024 = 35,159 genomes were used to infer a reference phylogeny from the bacterial dataset. Setting Δ = 0.7 correspond to a 5-fold reduction in the bacterial dataset, which represented a good compromise between the number of leaves in the reference tree and its readability.

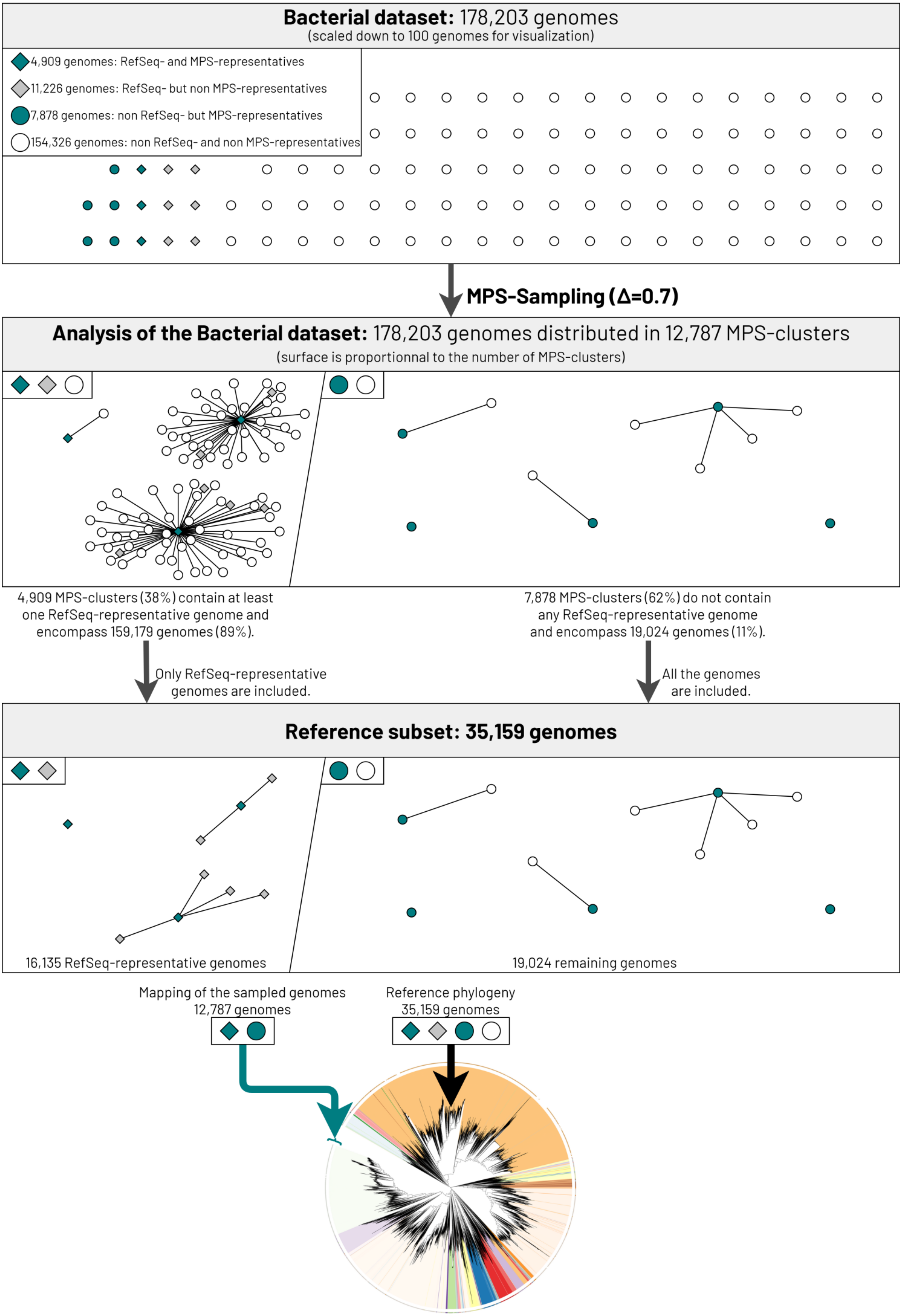
Bacterial dataset: 178,203 genomes
(scaled down to 100 genomes for visualization)
4,909 genomes: RefSeq- and MPS-representatives
11,226 genomes: RefSeq- but non MPS-representatives
7,878 genomes: non RefSeq- but MPS-representatives
154,326 genomes: non RefSeq- and non MPS-representatives
MPS-Sampling (Δ=0.7)
Analysis of the Bacterial dataset: 178,203 genomes distributed in 12,787 MPS-clusters
(surface is proportionnal to the number of MPS-clusters)
4,909 MPS-clusters (38%) contain at least one RefSeq-representative genome and encompass 159,179 genomes (89%).
7,878 MPS-clusters (62%) do not contain any RefSeq-representative genome and encompass 19,024 genomes (11%).
Only RefSeq-representative genomes are included.
All the genomes are included.
Reference subset: 35,159 genomes
16,135 RefSeq-representative genomes
19,024 remaining genomes
Mapping of the sampled genomes
12,787 genomes
Reference phylogeny
35,159 genomes

Additional file 31 – Monitoring the dereplication process of the bacterial dataset

A: Taxonomic redundancy of the samples. Average number of genomes representing each taxonomic level in the samples.

B: Genomic reduction of the 135,315 genomes and the 42,888 genomes with a complete and incomplete taxonomic affiliation, respectively, with a normalized scale.

C: Phylogenetic diversity of three subsets: the bacterial dataset (black), the subset of genomes with complete taxonomic affiliation (dark green) and the subset of genomes with incomplete taxonomic affiliation (orange). The phylogenetic diversity was computed by the length of all branches divided by the number of leaves.

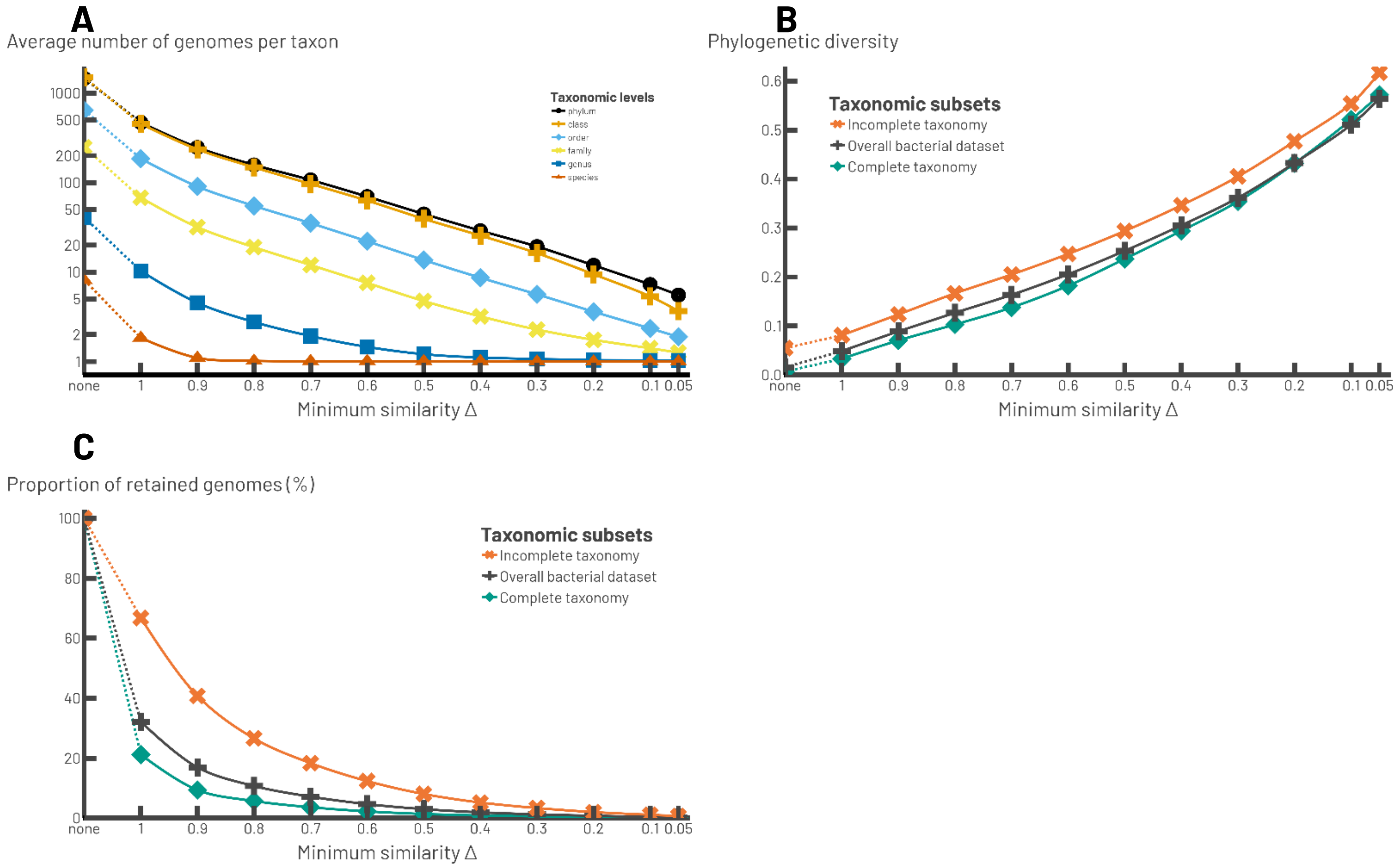

Additional file 32 – Monitoring the dereplication process of the GTDB dataset

MPS-Sampling was also tested on the data of the GTDB, encompassing 120 core protein families present in 394,932 bacterial genomes [14]. Here are some results.

A: Size of the samples built by MPS-Sampling.

B: Genomic reduction of the 135,315 genomes and the 42,888 genomes with a complete and incomplete taxonomic affiliation, respectively, with a normalized scale.

C: Taxonomic diversity of the samples. The proportion of phyla, classes, orders, families, genera, and species represented in each sample is indicated.

D: Taxonomic redundancy of the samples. Average number of genomes representing each taxonomic level in the samples.

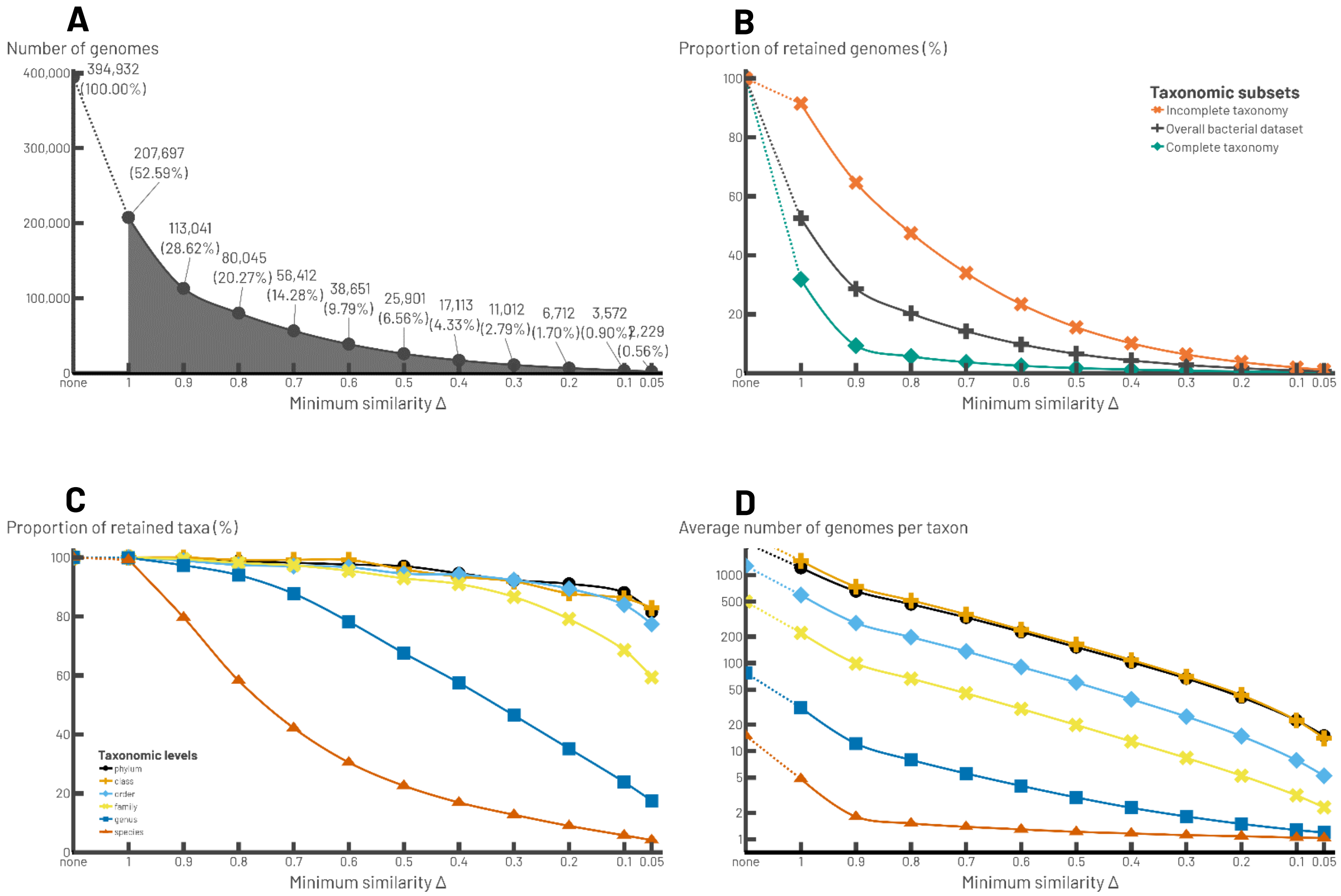

Additional file 33 – Sampling of *Lactobacillaceae*, *Bacillaceae* and *Enterobacteriaceae*

Sampling of *Lactobacillaceae* family (*Firmicutes* phylum)

The *Lactobacillaceae* family included 6,401 genomes from 33 genera and 379 species. The taxonomic distribution was balanced, with on average 17 genomes per species. Because the *Lactobacillaceae* was well studied and characterized, a high level of redundancy was observed at both taxonomic and genetic levels: the average number of genomes per genus and species was more than four times higher in this taxonomic family than in the complete bacterial dataset: 194 and 41 genomes per genus, and 17 and 8 genomes per species, respectively. This redundancy was also obvious at the phylogenetic level, as the diversity of *Lactobacillaceae* was 3 times lower than for the bacterial dataset (0.0047 and 0.0159, respectively) (Additional file 34B).

Consistently, the dereplication of *Lactobacillaceae* genomes was more intense than for *Bacteria* (Additional file 34A). For instance, when Δ = 1, 100% of the genera and 96% of the species were conserved, only 17% of the genomes were kept. As Δ decreased, the number of genomes per genus and per species gradually decreased to 1, meaning that all the intra-species and intra-genus redundancy was eliminated.

Compared to *Bacteria*, the reduction of *Lactobacillaceae* was 2 to 8 times higher (Additional file 34A), while the genetic diversity of the *Lactobacillaceae* gradually increased to reach a comparable level when Δ = 0.4 (Additional file 34B). The mapping of MPS-representatives on the phylogeny of *Lactobacillaceae* was also reliable, with a denser sampling in regions of the tree with greater phylogenetic diversity (**Erreur ! Source du renvoi introuvable.**-A1).This illustrated the ability of MPS-Sampling to adjust sampling intensity according to taxonomic and genetic redundancy in the data, but also to homogenize the taxonomic and genetic diversity of a data set at different evolutionary scales.

Sampling of *Bacillaceae* family (*Firmicutes* phylum)

The case of the *Bacillaceae* was more complex¸ because this family may not be monophyletic [15]. In this context, it was quite possible that part of the reconstructed MPS-clusters mixed genomes from *Bacillaceae* and other families, meaning that some *Bacillaceae* genomes may be represented by non-*Bacillaceae* genomes, and conversely. In the bacterial dataset, the *Bacillaceae* family included 7,113 genomes from 108 genera and 644 species. However, their taxonomic distribution was unbalanced (**Erreur ! Source du renvoi introuvable.**-B1), with the genus *Bacillus* alone comprising three-quarters of the *Bacillaceae* genomes (5,260 genomes out of 7,113). Despite this, the samples were reliable regarding the phylogenetic distribution of the MPS-representatives (**Erreur ! Source du renvoi introuvable.**-B1). In particular, the overrepresentation of *Bacillus* was successfully reduced as they account for 18% to 10% of the sampled genomes, 44 out of 249 when Δ = 0.7 and 4 out of 39 when Δ = 0.4, respectively. In fact, most MPS-representatives belonged to genera other than *Bacillus*, demonstrating the ability of MPS-Sampling to capture the genetic diversity of *Bacillaceae*. It also balanced the representativeness of *Bacillaceae*, whose taxonomy and genetic diversity in the samples became comparable to that of *Lactobacillaceae* and *Bacteria* (Additional file 34B).

Through the example of *Bacillaceae*, MPS-Sampling showed its ability to produce relevant samples even in cases where the initial level of redundancy was very high and unbalanced, but also when taxonomy and phylogeny disagreed.

Sampling of the *Enterobacteriaceae* family (*Proteobacteria* phylum)

The *Enterobacteriaceae* represented another interesting case because their taxonomy was more unbalanced than that of the *Bacillaceae*. The *Enterobacteriaceae* family included 17,096 genomes from 178 species and 58 genera, which theoretically corresponded to an average of 88 and 294 genomes per species and per genus, respectively). However, this was far from the case as six genera (*Klebsiella*,

Enterobacter, *Salmonella*, *Shigella*, *Escherichia*, and *Citrobacter)* accounted for 92% of the genomes (15,728 out of 17,096). Consistently, even with the densest sampling (Δ = 1), a few genomes (3.99%) were kept, compared to 32.17%, 17.07%, and 22.80% for *Bacteria*, *Lactobacillaceae*, and *Bacillaceae*, respectively (Additional file 34A). The situation was even more extreme when Δ = 0.4: as 0.11% of the genomes were retained, compared to 1.95%, 0.19%, and 0.55% for *Bacteria*, *Lactobacillaceae*, and *Bacillaceae*, respectively (Additional file 34A). When Δ ≤ 0.7, the six most represented genera were reduced to only 1 MPS-representative. Regarding the taxonomic density, one genome per species and genus was kept for *Enterobacteriaceae*, from Δ ≤ 0.9 to Δ ≤ 0.7 respectively, indicating that as for *Bacteria*, *Lactobacillaceae*, and *Bacillaceae*, most of the redundancy within genera and species was eliminated. However, dereplication was much higher, as 37.93% of the genera were conserved when Δ = 0.7, compared to 96.97%, and 86.11% for *Lactobacillaceae*, and *Bacillaceae*, respectively. This reflected a much lower inter-genera and inter-species diversity in *Enterobacteriaceae* than in the two other families. This might be due to differences in the delineation of taxa, which might reflect historical legacy and/or practical convenience, as in the case of the genera *Shigella* and *Escherichia* [16]. Regardless of the origin of these biases, taxonomy-based sampling would have led to an over-representation of *Enterobacteriaceae*, but also of higher taxa (i.e. *Enterobacterales* and *Gammaproteobacteria*) in the samples.

Considering phylogenetic diversity, *Enterobacteriaceae* were initially less diversified than *Bacteria*, *Lactobacillaceae*, and *Bacillaceae* (0.0008, compared to 0.0159, 0.0026, and 0.0046, respectively (Additional file 34B). As with other taxonomic groups, the phylogenetic diversity of *Enterobacteriaceae* increased as sampling density decreased. More precisely, although *Enterobacteriaceae* has the lowest initial phylogenetic diversity, the diversity of its MPS-samples is higher than for the other groups from Δ = 0.8 to Δ = 0.4 (Additional file 34B). This indicated that the true phylogenetic diversity of *Enterobacteriaceae* was hidden by its extreme genetic redundancy. And indeed, the *Enterobacteriaceae* included highly divergent strains and species, with very diverse lifestyles and habitats (e.g., insect endosymbionts, plant and animal pathogens, extremophiles…), some of which might have high rates of evolution. While being phylogenetically agnostic, but thanks to its ability to adapt the sampling density, MPS-Sampling succeeded in revealing the true phylogenetic diversity of *Enterobacteriaceae*, despite a very high initial redundancy.

Additional file 34 – Reduction of three taxonomic families

A: Genomic reduction of the three taxonomic families *Lactobacillaceae*, *Bacillaceae* and *Enterobacteriaceae*, in comparison with *Bacteria*.

B: Phylogenetic diversity of the three taxonomic families *Lactobacillaceae*, *Bacillaceae* and *Enterobacteriaceae*, in comparison with *Bacteria*, computed by the length of all branches divided by the number of leaves,

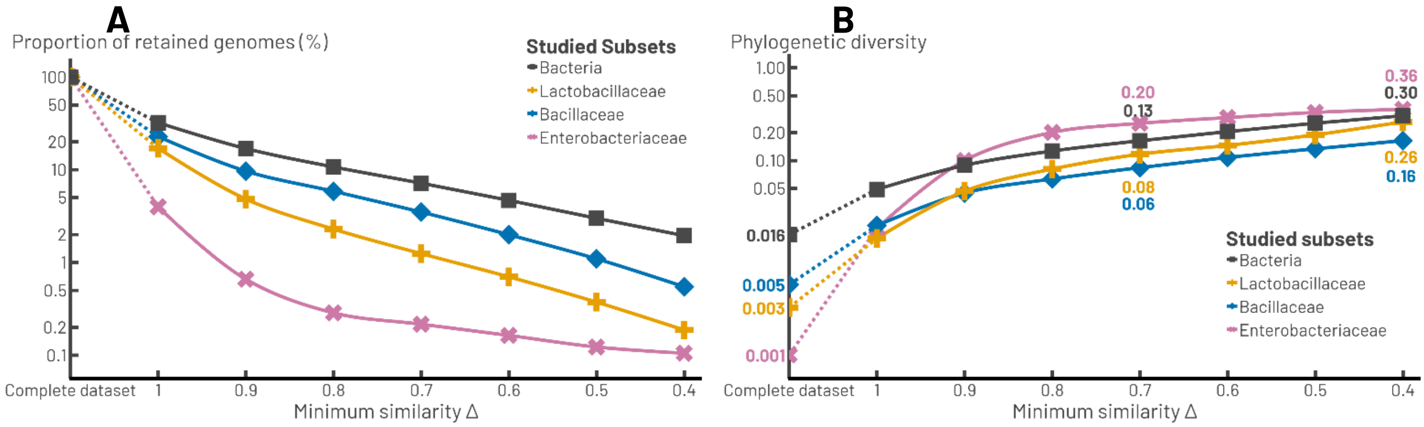

Additional file 35 – Phylogenetic mapping of MPS-representatives for three bacterial families

Phylogenetic mapping of MPS-representatives of three bacterial families: *Lactobacillaceae*, *Bacillaceae*, *Enterobacteriaceae* (Δ ∈ {0.7; 0.6; 0.5; 0.4})

A: ML tree of the 6,410 *Lactobacillaceae* genomes present in the bacterial dataset. The tree has been inferred with r-prot sequences. From the innermost to the outermost circle: MPS-representatives corresponding to Δ=0.7 (80 MPS-representatives), Δ=0.6 (45 MPS-representatives), Δ=0.5 (24 MPS-representatives), and Δ=0.4 (12 MPS-representatives). Colors correspond to the 33 genera of *Lactobacillaceae*.

B: ML tree of the 7,113 *Bacillaceae* genomes present in the bacterial dataset. The tree has been inferred with r-prot sequences. From the innermost to the outermost circle: MPS-representatives corresponding to Δ=0.7 (249 MPS-representatives), Δ=0.6 (142 MPS-representatives), Δ=0.5 (78 MPS-representatives), and Δ=0.4 (39 MPS-representatives). Colors correspond to the 108 genera of *Bacillaceae*.

C1: ML tree of the 17,096 *Enterobacteriaceae* genomes present in the bacterial dataset. The tree has been inferred with r-prot sequences. From the innermost to the outermost circle: MPS-representatives corresponding to Δ=0.7 (37 MPS-representatives), Δ=0.6 (28 MPS-representatives), Δ=0.5 (21 MPS-representatives), and Δ=0.4 (18 MPS-representatives). Colors correspond to the 58 genera of *Enterobacteriaceae*.

C2: ML tree of the 17,096 *Enterobacteriaceae* genomes with 16,987 leaves collapsed. It highlights the 18 Candidatus genera contained within the *Enterobacteriaceae* genomes, representing the large majority of the MPS-samples: 27 out of 37 and 14 out of 18 MPS-representatives are Candidatus genomes, for Δ=0.7 and Δ=0.4 respectively.

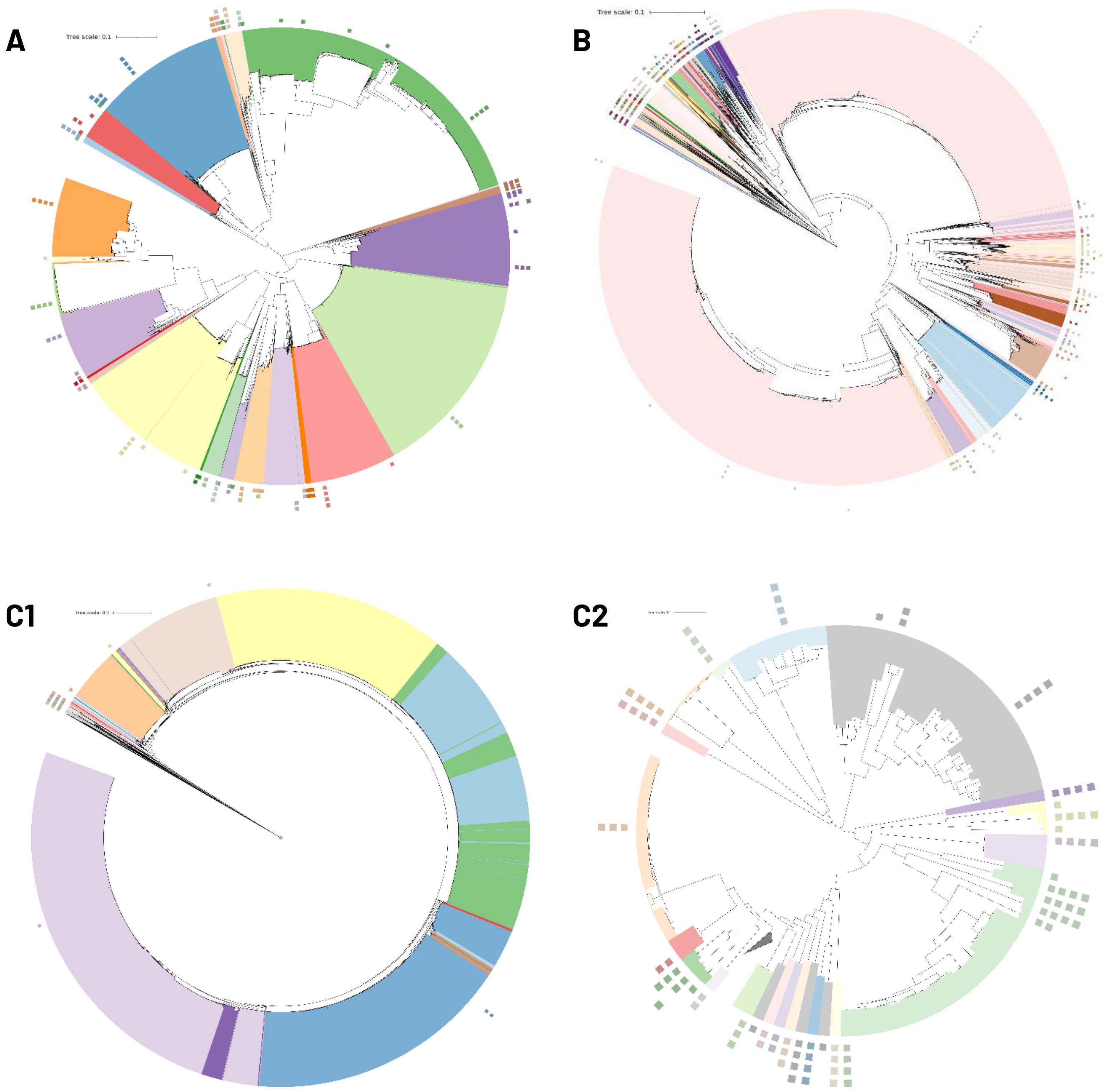

Additional file 36 – Phylogenetic mapping for *Enterobacteriaceae*

(legend on the next page)

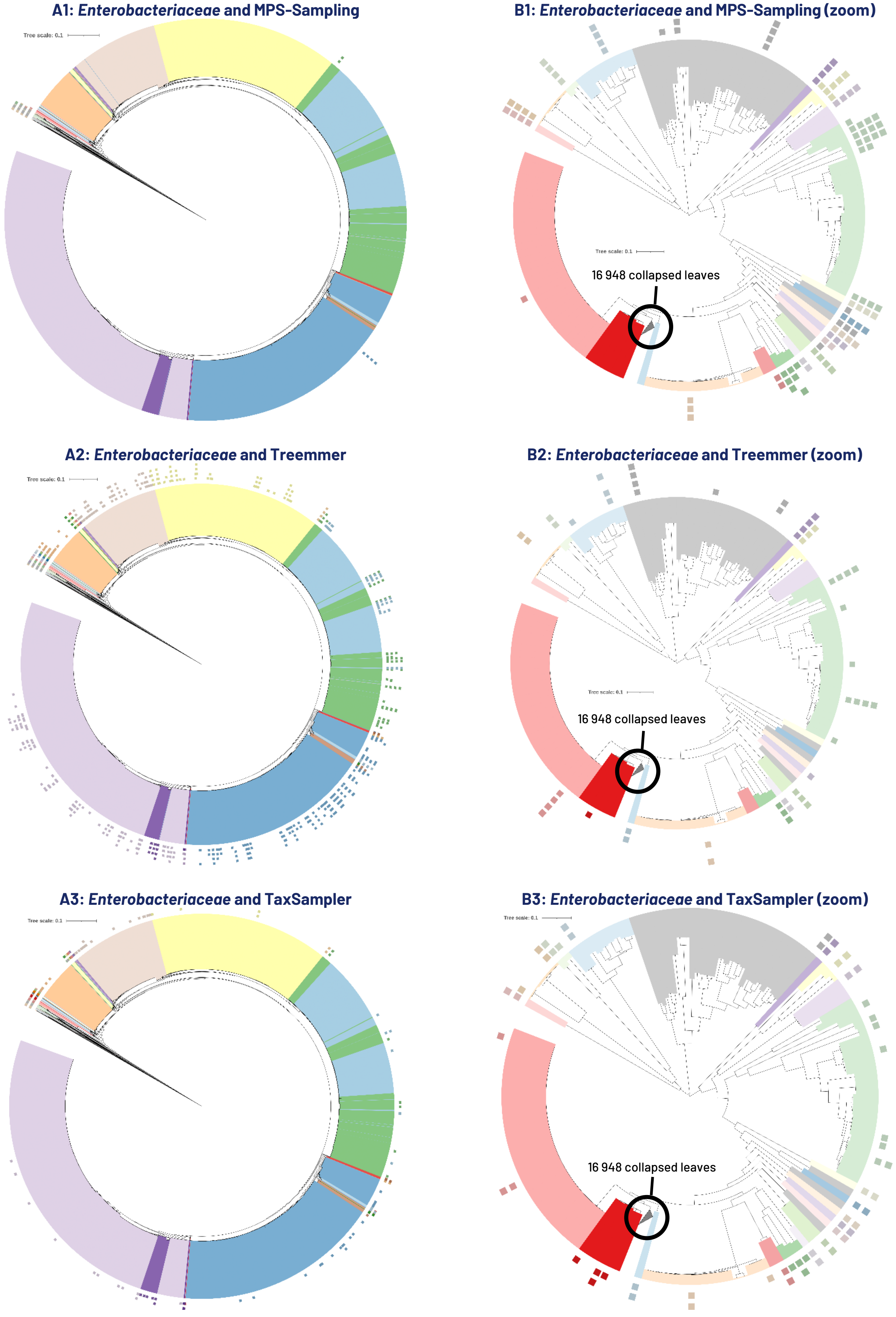

Sampling by the three methods (MPS-Sampling, Treemmer and TaxSampler) are compared for the taxonomic family *Enterobacteriaceae*.

A: Mapping of the MPS Sampling, Treemmer, and TaxSampler samples of *Enterobacteriaceae* on the phylogeny of the 17,096 genomes of *Enterobacteriaceae* contained in the bacterial dataset.

- **A1:** Mapping of the 36, 28, 21 and 18 genomes of *Enterobacteriaceae* present in the samples of the bacterial dataset obtained with MPS Sampling when $\Delta \in \{0.7; 0.6; 0.5; 0.4\}$.
- **A2:** Mapping of the 280, 195, 130 and 84 genomes of *Enterobacteriaceae* present in the Treemmer samples of the bacterial dataset of equivalent size to those obtained with MPS-Sampling when $\Delta \in \{0.7; 0.6; 0.5; 0.4\}$.
- **A3:** Mapping of the representatives of the 204 species and the 58 genera of *Enterobacteriaceae* selected by TaxSampler.

B: Same tree than in **(A)** but with 16,948 collapsed leaves (36 genera), in the small triangle at bottom left.

- **B1:** This triangle contains 2, 2, 1 and 1 MPS-representatives respectively.
  The rest of the tree therefore contains 34, 26, 20 and 17 MPS representatives respectively.
- **B2:** This triangle contains 263, 181, 119 and 77-Treemmer representatives respectively.
  - The rest of the tree therefore contains: 17, 14, 11 and 7 Treemmer representatives respectively.
- **B3:** This triangle contains respectively: 168 and 36 Tax-representatives.
  - The rest of the tree therefore contains: 36 and 22 Tax-representatives respectively.

References

1. O'Leary NA, Wright MW, Brister JR, Ciufo S, Haddad D, McVeigh R, et al. Reference sequence (RefSeq) database at NCBI: Current status, taxonomic expansion, and functional annotation. Nucleic Acids Res. 2016;44:D733–45.

2. Bateman A, Martin MJ, O'Donovan C, Magrane M, Alpi E, Antunes R, et al. UniProt: The universal protein knowledgebase. Nucleic Acids Res. 2017;45:D158–69.

3. Yates AD, Allen J, Amode RM, Azov AG, Barba M, Becerra A, et al. Ensembl Genomes 2022: An expanding genome resource for non-vertebrates. Nucleic Acids Res. 2022;50:D996–1003.

4. Köster J, Rahmann S. Snakemake-a scalable bioinformatics workflow engine. Bioinformatics. 2012;28:2520–2.

5. Anaconda Documentation. Anaconda Software Distribution. 2020. https://docs.anaconda.com/.

6. Steinegger M, Söding J. Clustering huge protein sequence sets in linear time. Nat Commun. 2018;9.

7. Steinegger M, Söding J. MMseqs2 enables sensitive protein sequence searching for the analysis of massive data sets. Nat Biotechnol. 2017;35:1026–8.

8. Song I, Evans M, Park EK. A Comparative Analysis of Entity-Relationship Diagrams. J Comput Softw Eng. 1995;3:427–59.

9. Harris D, Harris S. Digital Design and Computer Architecture. 2012.

10. Jauffrit F, Penel S, Delmotte S, Rey C, De Vienne DM, Gouy M, et al. RiboDB Database: A Comprehensive Resource for Prokaryotic Systematics. Mol Biol Evol. 2016;33:2170–2.

11. Lassmann T. Kalign 3: Multiple sequence alignment of large datasets. Bioinformatics. 2020;36:1928–9.

12. Philippe H, De Vienne DM, Ranwez V, Roure B, Baurain D, Delsuc F. Pitfalls in supermatrix phylogenomics. Eur J Taxon. 2017;2017:1–25.

13. Price MN, Dehal PS, Arkin AP. FastTree 2 - Approximately maximum-likelihood trees for large alignments. PLoS One. 2010;5.

14. Parks DH, Chuvochina M, Rinke C, Mussig AJ, Chaumeil PA, Hugenholtz P. GTDB: An ongoing census of bacterial and archaeal diversity through a phylogenetically consistent, rank normalized and complete genome-based taxonomy. Nucleic Acids Res. 2022;50:D785–94.

15. Maayer P De, Aliyu H, Cowan DA. Reorganising the order Bacillales through phylogenomics. Syst Appl Microbiol. 2019;42:178–89.

16. Lan R, Reeves PR. Escherichia coli in disguise: Molecular origins of Shigella. Microbes Infect. 2002;4:1125–32.
